# Endothelial colony-forming cell-derived exosomal miR-21-5p regulates autophagic flux to promote vascular endothelial repair by inhibiting SIPL1A2 in atherosclerosis

**DOI:** 10.1186/s12964-022-00828-0

**Published:** 2022-03-12

**Authors:** Xiao Ke, Zhiyong Liao, Xinlin Luo, Jun-qiu Chen, Ming Deng, Yiteng Huang, Zanxin Wang, Minxin Wei

**Affiliations:** 1 Department of Cardiology, Fuwai Hospital, Chinese Academy of Medical Sciences (Shenzhen Sun Yat-Sen Cardiovascular Hospital), Shenzhen, 518057 China; 2 Shenzhen University School of Medicine and Shenzhen University Health Science Center, Shenzhen, China; 3 Cardiac Surgery Department, The University of Hong Kong-Shenzhen Hospital, Shenzhen, China

**Keywords:** Atherosclerosis, Endothelial progenitor cell-derived exosomes, miR-21-5p, SIPA1L2, Autophagic flux

## Abstract

**Background:**

Percutaneous transluminal coronary angioplasty (PTCA) represents an efficient therapeutic method for atherosclerosis but conveys a risk of causing restenosis. Endothelial colony-forming cell-derived exosomes (ECFC-exosomes) are important mediators during vascular repair. This study aimed to investigate the therapeutic effects of ECFC-exosomes in a rat model of atherosclerosis and to explore the molecular mechanisms underlying the ECFC-exosome-mediated effects on ox-LDL-induced endothelial injury.

**Methods:**

The effect of ECFC-exosome-mediated autophagy on ox-LDL-induced human microvascular endothelial cell (HMEC) injury was examined by cell counting kit-8 assay, scratch wound assay, tube formation assay, western blot and the Ad-mCherry-GFP-LC3B system. RNA-sequencing assays, bioinformatic analysis and dual-luciferase reporter assays were performed to confirm the interaction between the miR-21-5p abundance of ECFC-exosomes and *SIPA1L2* in HMECs. The role and underlying mechanism of ECFC-exosomes in endothelial repair were explored using a high-fat diet combined with balloon injury to establish an atherosclerotic rat model of vascular injury. Evans blue staining, haematoxylin and eosin staining and western blotting were used to evaluate vascular injury.

**Results:**

ECFC-exosomes were incorporated into HMECs and promoted HMEC proliferation, migration and tube formation by repairing autophagic flux and enhancing autophagic activity. Subsequently, we demonstrated that miR-21-5p, which is abundant in ECFC-exosomes, binds to the 3’ untranslated region of SIPA1L2 to inhibit its expression, and knockout of miR-21-5p in ECFC-exosomes reversed ECFC-exosome-decreased SIPA1L2 expression in ox-LDL-induced HMEC injury. Knockdown of SIPA1L2 repaired autophagic flux and enhanced autophagic activity to promote cell proliferation in ox-LDL-treated HMECs. ECFC-exosome treatment attenuated vascular endothelial injury, regulated lipid balance and activated autophagy in an atherogenic rat model of vascular injury, whereas these effects were eliminated with ECFC-exosomes with knockdown of miR-21-5p.

**Conclusions:**

Our study demonstrated that ECFC-exosomes protect against atherosclerosis- or PTCA-induced vascular injury by rescuing autophagic flux and inhibiting SIAP1L2 expression through delivery of miR-21-5p.

**Video Abstract**.

**Supplementary Information:**

The online version contains supplementary material available at 10.1186/s12964-022-00828-0.

## Background

Atherosclerosis is one of the most common causes of death in the world, with a prevalence of 19 (males) or 14 (females) per 100,000 [1]. Atherosclerosis is primarily caused by the elevation of low-density lipoprotein cholesterol (LDL-c) with the primary symptom of intimal hyperplasia owing to endothelial injury, hyperproliferation of smooth muscle cells, and lymphocytic infiltration. Percutaneous transluminal coronary angioplasty (PTCA), known as an efficient therapeutic method against atherosclerosis, has significantly improved patient survival rates [2]. However, postsurgical restenosis may seriously interfere with patient prognosis, urgently requiring a solution [3, 4]. Therefore, developing a better method to restore the structural and functional integrity of blood vessels is of great importance for improving the prognosis of patients with atherosclerosis.

Endothelial progenitor cells (EPCs) are multipotential stem cells that differentiate into mature endothelial cells [5, 6]. Recent studies have shown that there are at least two subsets of EPCs, circulating angiogenic cells (CACs) and endothelial colony-forming cells (ECFCs), and ECFCs are considered true EPCs due to their capability to integrate directly into developing vessels and form tube-like structures in vitro, providing promising therapeutic potential [7]. Previous studies have revealed that EPCs may facilitate vascular reendothelialization and reduce intimal hyperplasia when transplanted into the injury site of carotid arteries [8–11]. However, abnormal differentiation, thrombosis, and immunogenicity impede the application of EPC transplantation; thus, an optimized approach is urgently needed. Recently, it has been shown that paracrine signalling plays a crucial role in EPC-mediated vascular repair [12], and the master paracrine product of EPCs is exosomes [13–15]. Hu et al. [16] reported that exosomes from umbilical cord blood (UCB) generated by EPCs improved the repair of balloon-induced mechanical vascular injury in a rat model. However, the difference between balloon-induced vascular injury and atherosclerosis, which is usually induced by lipid accumulation, may lead to varied outcomes and requires specific treatment.

Exosomes are highly efficient in intercellular communication, as they are capable of transmitting biodegradable molecules in a protective manner. Emerging evidence has shown that paracrine exosomes secreted by different cells play crucial roles in atherosclerosis via their protein and noncoding RNA (such as miRNA) components, which may become therapeutic approaches for atherosclerosis [17–19]. Zhu et al. [20] reported that nicotine-stimulated macrophage-derived exosomes accelerate atherosclerosis through miR-21-3p/PTEN-mediated VSMC migration and proliferation. Bouchareychas et al. [21] indicated that exosome delivered anti-inflammatory miR-99a/146b/378a from bone marrow-derived macrophages to resolve atherosclerosis by regulating haematopoiesis and inflammation by targeting NF-κB and TNF-α signalling. Yang et al. [22] demonstrated that exosomes from mesenchymal stem cells efficiently delivered miR-145 to endothelial cells and inhibited atherosclerosis by targeting JAM-A. In addition, recent studies have indicated that EPC-derived exosomes protect against endothelial injury to promote vascular repair in balloon injury-induced vascular injury in rats with normal blood lipid levels [23–25]. However, the therapeutic effect and underlying mechanism of ECFC-exosomes on vascular injury induced by balloon injury in hyperlipidaemic rats remain unclear and require further examination.

In this study, we investigated the therapeutic effects of ECFC-exosomes in a rat model of atherosclerosis established by hyperlipidaemia with balloon-induced injury. The molecular mechanisms underlying the ECFC-exosome-mediated effects on ox-LDL-induced endothelial injury were also explored.

## Materials and methods

### Isolation and culture of ECFCs from human peripheral blood

All experimental procedures were approved by the Ethical Committee of the Fuwai Hospital Chinese Academy of Medical Sciences (NO.: SP2019002), and each patient signed informed consent. ECFCs were isolated from human peripheral blood as previously described [26]. In brief, human whole blood (50 mL) was collected from 8 healthy volunteers (aged between 30 and 40 years old). Blood samples were diluted in phosphate-buffered saline (PBS) at a 1:1 ratio and added onto separation medium (GE Healthcare, Pittsburgh, USA) with endothelial cell growth factor and cytokines. Thereafter, the blood samples were centrifuged at 1200*g* for 30 min, and the mononuclear cells were collected and washed with PBS. The cells were then placed into a 25 cm^2^ culture flasks with endothelial cell growth medium (EGM-2; Thermo Fisher Scientific, Waltham, USA) containing 10% foetal bovine serum (FBS, Gibco, Waltham, USA), 100 U/mL penicillin (Gibco) and 100 μg/mL streptomycin (Gibco). The FBS used in this study was centrifuged in advance using density gradient centrifugation to remove pre-existing exosomes. After 72 h, nonadherent cells were removed. The medium was refreshed every 3 days, and cells were cultured in a 5% CO_2_ humidified atmosphere at 37 °C. Cells at passages 3–6 were used in subsequent experiments. Cell morphology was examined under a light microscope following culturing for 0, 3, 7, 10 and 21 days. ECFCs were successfully isolated from the peripheral blood of 6 of the 8 healthy volunteers.

### Identification of human peripheral blood-derived ECFCs

For immunocytochemistry, cells were fixed in 4% paraformaldehyde for 15 min and permeabilized with 0.1% Triton X-100 for 10 min at room temperature. After blocking with 3% bovine serum albumin (BSA) for 1 h, cells were incubated with primary antibodies overnight at 4℃ and then incubated with secondary antibodies for 1 h at 37 °C. Nuclei were stained with 4,6-diamidino-2-phenylindole (DAPI; 0.5 µg/ml; Invitrogen, USA) for 5 min. Cells were washed and analysed using a fluorescence microscope (Leica, Germany). Antibodies, including anti-CD45, anti-CD144, anti-eNOS, anti-vWF and their respective secondary antibodies, were obtained from Abcam (Cambridge, UK). Flow cytometry analysis was performed using a BD Accuri™ C6 flow cytometer (BD Biosciences). Cells were stained with CD34-FITC, CD144-FITC, vWF-FITC and CD45-FITC antibodies using standard procedures and were then measured and analysed using a BD Accuri™ C6 flow cytometer.

### Assessment of acetylated low-density lipoprotein (ac-LDL) uptake and Ulex europaeus agglutinin-1 (UEA-l) binding

The uptake of DiL-labelled ac-LDL (Dil-ac-LDL; Molecular Probes, Eugene, USA) and binding of FITC-conjugated UEA-1 (FITC-UEA-l; Sigma–Aldrich, USA) by ECFCs were assessed by fluorescence staining. Briefly, cells were incubated with Dil-ac-LDL (15 µg/mL) for 4 h at 37 °C and fixed in 4% paraformaldehyde for 30 min. After washing, cells were stained with FITC-UEA-l (10 µg/mL) for 1 h at 37 °C and with DAPI for 5 min. Cells were washed and analysed using a fluorescence microscope (Leica DMI6000B, Germany).

### Preparation and identification of exosomes from human peripheral blood-derived ECFC-exosomes

ECFCs were cultured in complete medium until reaching 80% confluence. The medium was then replaced with EGM-2 medium supplemented with 1 × serum replacement solution (PeproTech, Rocky Hill, USA). After incubation for an additional 48 h, the conditioned medium of ECFCs was collected and centrifuged at 300*g* for 20 min and 2000*g* for 10 min at 4 °C to remove dead cells and cellular debris. Thereafter, the supernatant was filtered through a 0.22 μm filter (Millipore, Burlington, USA) followed by centrifugation at 10,000*g* for 30 min. The pellet was discarded, and the supernatant was centrifuged at 100,000*g* for 70 min. The pellet was resuspended in PBS and centrifuged at 100,000*g* for 70 min and then resuspended again in PBS and stored at − 80 °C.

Total protein concentration of the exosomes was measured using a BCA protein assay (Pierce, T6hermo Scientific). The exosomes were characterized by morphologic examination using a transmission electron microscope (Hitachi H-7650; Japan), and the images were captured using a digital camera (Olympus). Western blots were conducted to detect protein levels of CD31, CD63, CD9 and CD81 in exosomes. The size distribution and concentration of the exosomes were analysed using Nanosight (NTA).

### Culture and treatment of human microvascular endothelial cells (HMECs)

HMECs (Lonza, Basel, Switzerland) were cultured in endothelial cell medium with 5% foetal bovine serum, 1% endothelial cell growth supplement, and 1% penicillin/streptomycin solution (ScienCell Research Laboratories, Carlsbad, USA) at 37 °C under a 5% CO_2_ atmosphere. HMECs between passages 3–7 were used in this study. Cells were treated with or without ox-LDL for 1 h followed by treatment with 100 μg/mL ECFC-exosomes or an autophagy inhibitor (10 mM bafilomycin A1, Selleckchem) for another 24 h. In the ECFC-exosome treatment experiments, HMECs were cultured in MCDB131 medium without serum or growth factors.

### Exosome uptake by HMECs

ECFC-exosomes were labelled with Vybrant DiO dye (Molecular Probes, Carlsbad, CA, USA) according to the manufacturer's instructions. The labelled exosomes (8 μl) were incubated with HMECs at 37 °C for 2 h. HMECs were washed with PBS, fixed in 4% paraformaldehyde for 15 min, and stained with DAPI for 5 min at room temperature. After washing, the cells were analysed using a fluorescence microscope (Leica DMI6000B, Solms, Germany).

### miRNAs and shRNA transfection

The miR-21-5p mimics, miR-21-5p inhibitor, SIPA1L2 shRNAs and their negative controls (NC-mimics, NC-inhibitor, and sh-NC) were purchased from GenePharma (Shanghai, China). The transfection of miRNAs and SIPA1L2 shRNA into HMECs was performed using Lipofectamine 2000 reagent (Invitrogen) according to the manufacturer’s protocol. Transfection of miRNAs into ECFC-exosomes was performed using an Exo-Fect Exosome Transfection Kit (System Biosciences, Palo Alto, USA) according to the manufacturer’s protocol. The sequences of the miR-21-5p mimics, miR-21-5p inhibitor, SIPA1L2 shRNAs and their negative controls are shown in Additional file 1: Table S1.

### Cell counting kit-8 (CCK-8) assay

HMECs (5000 cells/well in 96-well plates) were adjusted to different treatments for 24 h at 37 °C followed by culture with 10 μl cell counting kit-8 (CCK-8) reagent (Abcam) in each well for 1 h at 37 °C. Cell viability was determined by measuring the optical density values at 450 nm using a microplate reader (Thermo Fisher Scientific).

### Lactate dehydrogenase (LDH) activity

Lactate dehydrogenase (LDH) activity in culture medium was determined using an LDH activity assay kit (Solarbio, China) following the manufacturer’s protocol. Briefly, the cell medium was collected, and then the medium was mixed with different reagents from the LDH activity assay kit. Finally, the absorbance of each sample was detected at 450 nm using a microplate reader. LDH activity (U/L) was calculated as follows: (OD_U _− OD_C_) × C_S_ × N × 1000/(OD_S_  − OD_B_), where OD_U_ represents the sample tube absorbance value, OD_C_ represents the absorbance value of the blank tube, C_S_ represents the standard concentration (2 mmol/L), N represents multiples of dilutions of samples before testing, OD_S_ represents the absorbance value of the standard tube, and OD_B_ represents the absorbance value of the control tube.

### Wound healing assay

HMECs with different treatments were seeded into 6-well plates. When the cells reached 80% confluence, the cultured HMECs were scratched using a 200-μl pipette tip to create a wound area. The migration distances of cells were imaged 0 h and 24 h after scraping on an Olympus IX-71 inverted microscope equipped with an Olympus camera and were analysed using ImageJ software. The percentage of wound closure was calculated by the following equation: wound closure (%) = (wound area at 0 h − wound area at 24 h) × 100/(wound area at 0 h).

### Tube formation assay

After the addition of Matrigel (BD Biosciences, Franklin Lakes, USA), 24-well plates were gently agitated and incubated at 37 °C to form a gel. HMECs (2 × 10^4^ cells/well) with different treatments were plated into coated wells and then cultured at 37 °C for 8 h. Finally, images of each sample were captured using an Olympus IX-71 inverted microscope equipped with an Olympus camera. The acquired images were analysed using the Angiogenesis Analyser tool in ImageJ software, measuring the number of meshes and tube length. The average number of meshes formed and the percent tube length were calculated as tube formation ability.

### Quantitative real-time PCR (qRT-PCR)

Total RNA from isolated exosomes, cells and tissues was extracted using TRIzol reagent (Invitrogen, Carlsbad, USA) according to the manufacturer’s protocol. The quality of RNA was evaluated using a microspectrophotometer (NanoDrop, Wilmington, USA). For miRNA, qRT-PCR was conducted using the Hairpin-it™ miRNAs qPCR Quantitation Assay Kit (GenePharma) with U6 as an internal control. For mRNA, qRT-PCR was performed using the PrimeScript™ RT reagent Kit with gDNA Eraser (Takara, Dalian, China) and SYBR Premix Ex TaqTM Kit (Takara) with GAPDH as the internal control. Real-time PCRs were performed in a CFX96 Real-Time System thermocycler (Bio-Rad, Hercules, USA). The relative expression of genes was calculated using the comparative Ct method. All primer sequences are listed in Additional file 1: Table S1.

### Western blot

The exosomes, cells and tissues collected after treatment were lysed and homogenized in RIPA lysis buffer (Beyotime, Beijing, China). After centrifugation at 8000*g* for 10 min, the samples were quantified using the BCA method, denatured at 95 °C, and loaded at equal amounts onto SDS–PAGE gels. The proteins were resolved by electrophoresis and then transferred to PVDF membranes (Millipore, Burlington, USA). Subsequently, the membranes were blocked with 5% non-fat milk at room temperature for 1 h and incubated with primary antibodies, including anti-CD63 (#ab134045, Abcam), anti-CD9 (#ab92726, Abcam), anti-CD81 (#ab79559, Abcam), anti-CD31 (#ab28364, Abcam), anti-LC3 (#ab192890, Abcam), anti-p62 (#ab56416, Abcam), anti-Beclin1 (#ab207612, Abcam), anti-SIPA1L2 (#PA5-20848, Thermo Fisher Scientific) and anti-β-actin (#ab8226, Abcam), at 4 °C overnight. Following incubation with horseradish peroxidase-conjugated secondary antibodies, the immunoactivities were visualized using an ECL kit (GE healthcare) and semiquantified using ImageJ software (https://imagej.nih.gov/ij/).

### Tracking autophagy with double-tagged LC3B

Tracking autophagy using double-tagged LC3B was previously illustrated in detail [27]. Briefly, HMECs were infected with Ad-mCherry-GFP-LC3B (Beyotime) followed by different treatments. Then, the HMECs were stained with Hoechst 33342. Finally, autophagy was evaluated by the detection of mCherry and GFP using fluorescence microscopy (Leica). When autophagy occurs, mCherry-GFP-LC3B aggregates on the autophagosome membrane, presenting as yellow dots. When the autophagosome and lysosome fuse, they present as red spots due to partial quenching of GFP fluorescence, indicating smooth autophagic efflux. Each sample was assessed using three to five randomly selected fields under a fluorescence microscope (Leica), and at least 10 cells from each field were randomly selected for autophagy analysis.

### Expression profiling analysis of miRNA and mRNA

A schematic diagram of biological sample processing before miRNA and mRNA expression profiling is shown in Additional file 2: Figure S1.

For miRNA expression profile analysis in ECFC exosomes, we performed miRNA microarray analysis. In brief, total RNA was extracted from ECFC-exosomes using TRIzol reagent (Invitrogen). Subsequently, total RNA was labelled using a FlashTag Biotin HSR RNA Labeling Kit (Affymetrix, USA) following the manufacturer’s protocol and then hybridized with a GeneChip miRNA 4.0 Array (Affymetrix, USA). After hybridization, array images were digitized using a laser scanner interfaced with ArrayPro image analysis software (Media Cybernetics, Silver Spring, USA) to generate raw data. The obtained raw data were first normalized with robust multiarray average (RMA) using Expression Console software (version 1.3.1; Affymetrix, Inc.) and then analysed using Affymetrix Expression Console Software (version 1.3.1).

For mRNA expression profile analysis, total RNA was extracted from three independent samples of ECFC-exosomes + ox-LDL- or PBS + ox-LDL-treated HMECs using TRIzol reagent (Invitrogen) according to the manufacturer’s recommended protocol, and RNA quantity was assessed using a NanoDrop ND-2000 spectrophotometer (NanoDrop Technologies). After purifying mRNA using the RiboZero Magnetic Gold Kit, cDNA libraries were constructed for the KAPA Stranded RNA-Seq Library Prep kit (Illumina, Inc.) according to the manufacturer’s instructions. Subsequently, we used Agilent 2100 and qPCR to assess the quality and quantification of the cDNA library. Finally, RNA sequencing was performed using next-generation sequencing on an Illumina HiSeq Xten platform. Clean data were obtained from the raw data by removing reads containing adapters, reads containing more than 10% poly N, and low-quality reads and subsequently aligning the genome to the specified reference genome (Homo sapiens. GRCh38, NBCI) to obtain the mapped data. Differentially expressed mRNAs between ECFC-exosome-treated HMECs and PBS-treated HMECs were analysed using the EBseq R package, and fold changes (FCs) ≥ 2 and false discovery rates (FDRs) < 0.05 served as the screening criteria to identify differentially expressed mRNAs.

### Dual-luciferase reporter assay

The wild-type (WT) 3’ untranslated region (3’UTR) or mutant (MUT) 3’UTR sequences of *SIPA1L2* were cloned into pmirGLO (Promega). HMECs were cotransfected with plasmids containing WT 3’UTR or MUT 3’UTR sequences of *SIPA1L2* and miR-21-5p mimics or NC mimics using Lipofectamine 2000 (Invitrogen). After 48 h, luciferase activity was analysed using a Dual-Glo® Luciferase Assay System (Promega) and a MicroLumatPlus LB96 V luminometer (Berthold). Relative luciferase activity was calculated as firefly luciferase activity/Renilla luciferase activity.

### Atherogenic rat model of vascular injury

Male Sprague‐Dawley rats (200–250 g) were purchased from the Laboratory Animal Centre in Guangdong (Guangzhou, China). Animals were housed in a temperature‐controlled environment (21 ± 1 °C) with 40–60% humidity with a 12 h light/dark cycle and were provided free access to tap water and regular chow. The animal protocol was reviewed and approved by the Institutional Animal Care and Use Committee of Fuwai Hospital, Chinese Academy of Medical Sciences. All experiments were performed in strict accordance with the recommendations in the Guide for the Care and Use of Laboratory Animals of the National Institutes of Health. After 1 week of adaptive feeding, all rats were randomly divided into four groups: (a) Sham, (b) Model, (c) Exos-NC inh., and (d) Exos-miR inh. An atherogenic rat model of vascular injury was established by feeding high‐fat diets and inducing balloon injury as previously described [28, 29] with minor modifications. In brief, the rats were given a left carotid arterial intima injury surgically suing a 2F Fogarty arterial embolectomy balloon catheter after 4 weeks of high-fat feeding, and then the rats continued to be fed high-fat diets until the end of the study. High‐fat diets were composed of 81.5% basic diets, 10% lard, 0.5% sodium cholate, 3% cholesterol, and 5% sugar. The dose of high‐fat diets was 150 g/day. For the model group, rats received 100 µl PBS via tail vein injection after the surgery and were fed a high-fat diet throughout the experimental period; for the Exos-NC inh. group, the atherosclerotic rats received 100 μg ECFC-exosomes transfected with inhibitor NC after the surgery and were fed a high-fat diet throughout the experimental period; for the Exos-miR inh. group, the atherosclerotic rats received 100 μg ECFC-exosomes transfected with miR-21-5p inhibitor after the surgery and were fed a high-fat diet throughout the experimental period. For the sham group, the rats were injected with 100 µl PBS via tail vein injection after sham surgery and were fed a normal diet throughout the experimental period. The sham-operated rats were subjected to anaesthesia and surgical procedures without balloon injury. The rats were euthanized using an overdose of pentobarbital (80 mg/kg, i.p.) t Fourteen days after different treatments, the blood and carotid arteries were collected and processed as described below for further analysis.

### Evaluation of in vivo reendothelialization

Rats were intravenously injected with 5% Evans Blue dye (25 mg/kg) for 30 min before being sacrificed. The left common carotid artery was fully removed and rinsed in saline, and residual connective tissue was carefully removed. The staining area and the total area of the artery were analysed using Image-Pro Plus. Reendothelialization was evaluated by calculating the ratio of the staining area to the total area.

### Evaluation of intimal hyperplasia

Carotid arterial tissue sections were deparaffinized in xylene for 5 min, transferred to the dye solution in distilled water, stained with haematoxylin for 5 min, and then separated with a 1% hydrochloric acid alcohol in a saturated solution of lithium carbonate. After a short period of bluing, the sections were quickly washed in distilled water, stained with approximately 0.5% eosin dye solution for 1–3 min, and finally dehydrated using gradient alcohol. After the xylene became transparent, a suitable dose of neutral gum was added to seal the film. The degree of aortic pathology was observed using an optical microscope, and the samples were imaged. The sections were analysed using Image-Pro Plus 6.0 image processing software.

### Evaluation of serum lipid profiles

Rats were anaesthetized 1 day before surgery and 2 weeks after surgery, and blood samples were collected for serum lipid (triglyceride: TG, total cholesterol: TC, low-density lipoprotein cholesterol: LDL-C, high-density lipoprotein cholesterol: HDL-C) analysis. TG levels were measured using a TG Quantification Kit (Abcam, Cambridge, MA, USA) according to the manufacturer's instructions. TC levels were assessed using a Cholesterol Assay Kit (Abcam, Cambridge, MA, USA) according to the manufacturer's instructions. LDL-C levels were evaluated using an LDL-C Quantification Kit (Abcam, Cambridge, MA, USA) according to the manufacturer's instructions, and HDL-C levels were measured using an HDL-C Quantification Kit (Abcam, Cambridge, MA, USA) according to the manufacturer's instructions.

### Statistical analysis

Data are shown as the mean ± standard deviation (SD). Data analysis was performed using GraphPad Prism 6.0 (GraphPad Software, La Jolla, USA). Statistical significance for comparisons between two groups was analysed using the Mann–Whitney U test, and statistical significance for comparisons among more than two groups was analysed using the Kruskal–Wallis test followed by Dunn’s multiple comparisons test. *P* < 0.05 was considered statistically significant.

## Results

### Characterization of human PB-derived ECFCs and ECFC-exosomes

**Fig. 1:**
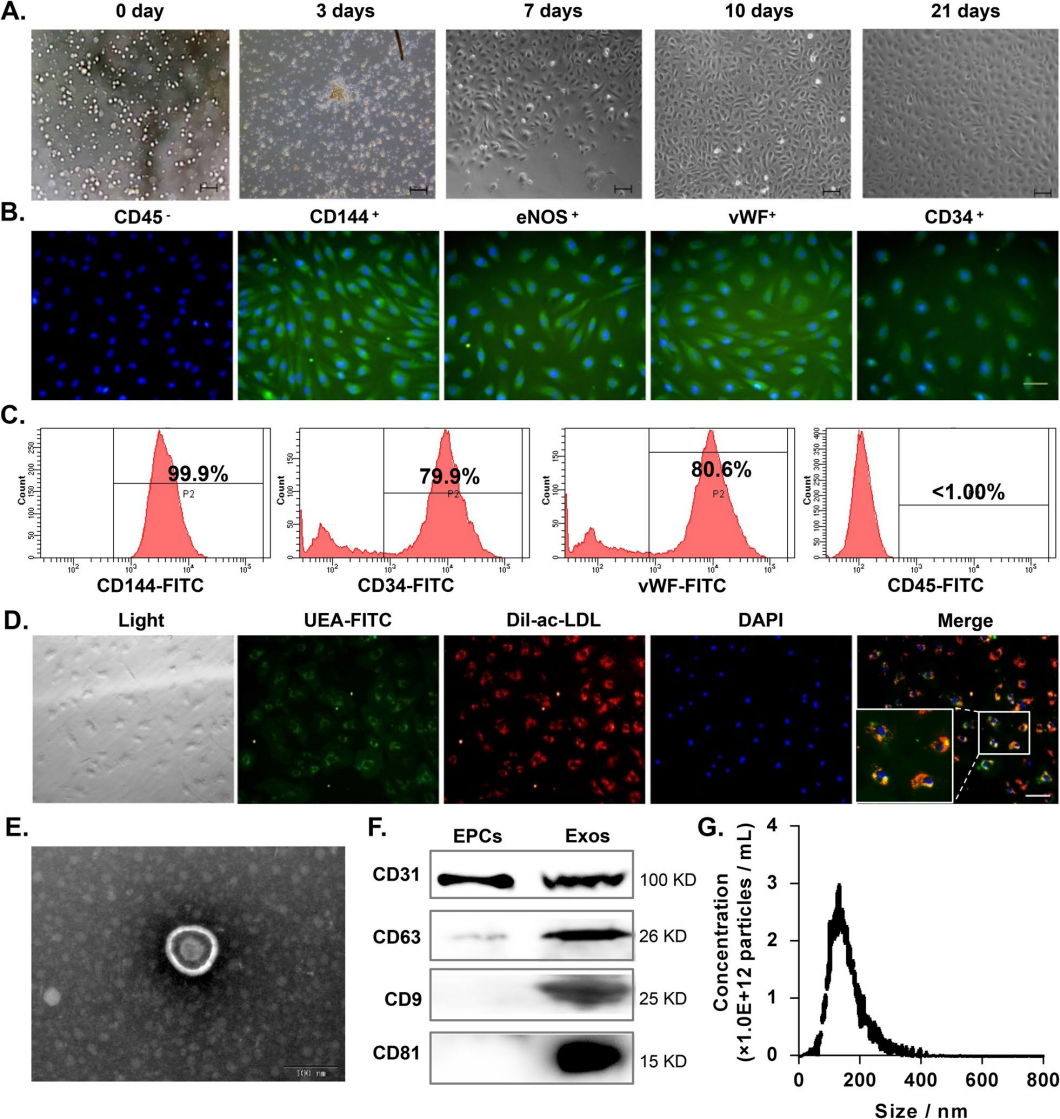
Characterization of human ECFCs. **A** Morphology of EPCs cultured for 0, 3, 7, 10 and 21 days. Scale bar = 100 μm. **B** Immunofluorescence staining of endothelial progenitor cell-specific surface markers (CD144, eNOS, vWF and CD34) and haematopoietic cell-specific markers (CD45) in the cells. Nuclei were counterstained using DAPI. Scale bar = 100 μm. **C** Flow cytometry analysis showing that EPCs were positive for CD144, CD34 and vWF but negative for the haematopoietic cell-specific marker CD45. **D** Immunofluorescence examination of ac-LDL (Dil) uptake and UEA-1 (FITC) binding capability of ECFCs. Nuclei were counterstained with DAPI. Scale bar = 50 μm. **E** The morphology of ECFC-derived exosomes was examined by TEM. Scale bar = 100 nm. **F** The surface markers CD63, CD9, and CD81 of exosomes were examined by western blot assay. *Exos* ECFCs-exosomes. **G** The particle sizes and concentrations of ECFC-exosomes were measured using NanoSight. Biological replicates = 3, and technical replicates = 1

ECFCs were isolated from human peripheral blood (PB) and cultured in vitro. PB-derived cells adhered within 3 days and proliferated for more than 21 days. Morphology of the isolated cells cultured at different time points is shown in Fig. 1A. Immunofluorescence staining revealed that PB-derived cells were positive for ECFC-specific surface markers (CD144, eNOS, vWF and CD34) but were negative for the haematopoietic cell-specific marker CD45 (Fig. 1B). The results of flow cytometry analysis also indicated that PB-derived cells were highly positive for numerous endothelial lineage markers (CD144, vWF and CD34) and were negative for CD45 (Fig. 1C). Furthermore, functional analysis using fluorescence tracking revealed that ECFCs were able to take up ac-LDL-c and bind to UEA-1 (Fig. 1D). These data demonstrated that we obtained high purity ECFCs. Next, ECFC-exosomes were purified from the supernatant of ECFC cultures and then identified by transmission electron microscopy (TEM), nanoparticle tracking analysis (NTA) and western blot analyses. TEM indicated that ECFC exosomes exhibited a typical sphere-shaped bilayer membrane morphology structure with a diameter of approximately 100 nm (Fig. 1E). Western blot results revealed that ECFC-exosomes were positive for exosome surface markers (CD63, CD9 and CD81) and the endothelial marker CD31, while ECFCs expressed only the endothelial marker CD31 (Fig. 1F). NTA demonstrated that the average diameter of ECFC-exosomes was 140.1 nm, and the main peak of particle size was located at 132.5 nm (Fig. 1G).

### ECFC-exosomes suppress ox-LDL-induced HMEC injury by rescuing autophagic flux

**Fig. 2:**
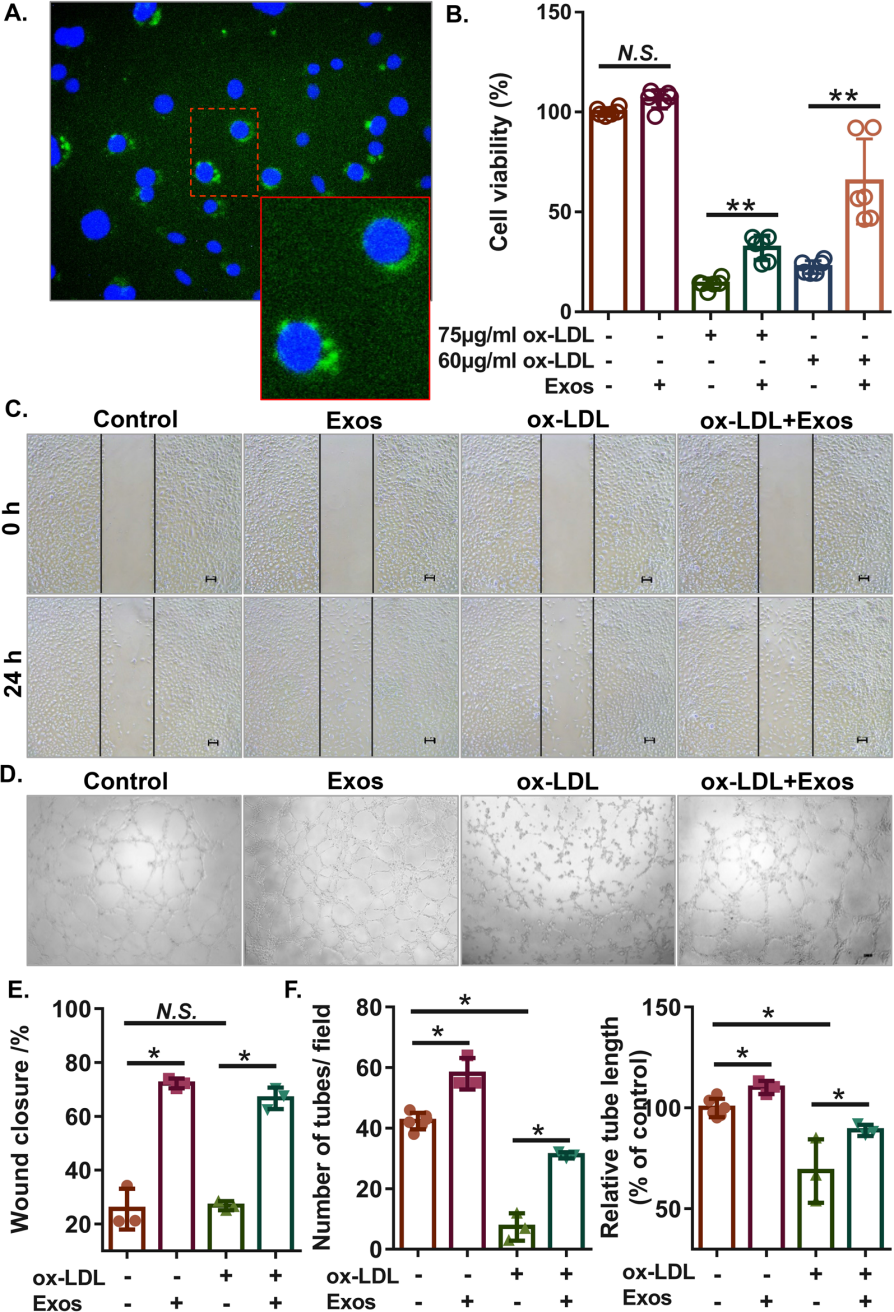
ECFC-derived exosomes promote the proliferation, migration, and tube formation of ox-LDL-treated HMECs. **A** Representative images of Dio-labelled ECFC-exosomes absorbed by HMECs. Red arrowheads indicated ECFCs-exosomes. Biological replicates = 3, and technical replicates = 1. **B** Cell viability of ox-LDL-treated HMECs with or without ECFC-exosome treatment was determined using CCK-8 assay. Biological replicates = 6, and technical replicates = 3. **C, E** Cell migration of ox-LDL-treated HMECs with or without ECFC-exosome treatment was determined by wound healing assay. Biological replicates = 3, and technical replicates = 1. **D, F** The tube formation ability of ox-LDL-treated HMECs with or without ECFC-exosome treatment was determined by tube formation assay. Scale bars = 100 μm. *Exos* ECFCs-exosomes. Biological replicates = 3–5, and technical replicates = 1. *N.S.* not significant; significant differences between different treatment groups are indicated as *P < 0.05 and **P < 0.01

To evaluate the role of ECFC-exosomes in protecting against vascular intima injury, an ox-LDL-induced HMEC injury model was established. First, HMECs were incubated with Dio-labelled ECFC-exosomes for 12 h, and then the green fluorescence signal of Dio in HMECs was observed under a fluorescence microscope, which was primarily distributed in the cytoplasm (Fig. 2A), suggesting that ECFC-exosomes were taken up by HMECs. Subsequently, to estimate the effect of ECFC-exosomes on cell viability, we treated HMECs with 100 µg/ml ECFC exosomes in the presence or absence of ox-LDL, in which ox-LDL was used to imitate the microenvironment of atherosclerosis in vitro. The CCK-8 results showed that ECFC-exosomes had no significant effect on the viability of HMECs without ox-LDL treatment, while they significantly reversed the viability of HMECs treated with 60 μg/mL or 75 μg/mL ox-LDL, and ECFC-exosome-mediated effects were more profound in HMECs treated with 60 μg/ml ox-LDL (Fig. 2B). Thus, 60 μg/ml ox-LDL was used in subsequent experiments. Moreover, ECFC-exosomes significantly increased the migration of HMECs without ox-LDL treatment. The migration of HMECs was not affected by 60 μg/ml ox-LDL treatment, while ECFC-exosome treatment significantly enhanced HMEC migration in ox-LDL-treated HMECs (Fig. 2C, E). In addition, ECFC-exosomes increased cell tube formation ability in HMECs without ox-LDL treatment, while tube formation ability of HMECs was significantly repressed by 60 μg/ml ox-LDL treatment, which was largely rescued by ECFC-exosome treatment (Fig. 2D, F). These results suggested that ECFC-derived exosomes enhance cell viability, migration and tubule formation in HMECs treated with ox-LDL.

**Fig. 3:**
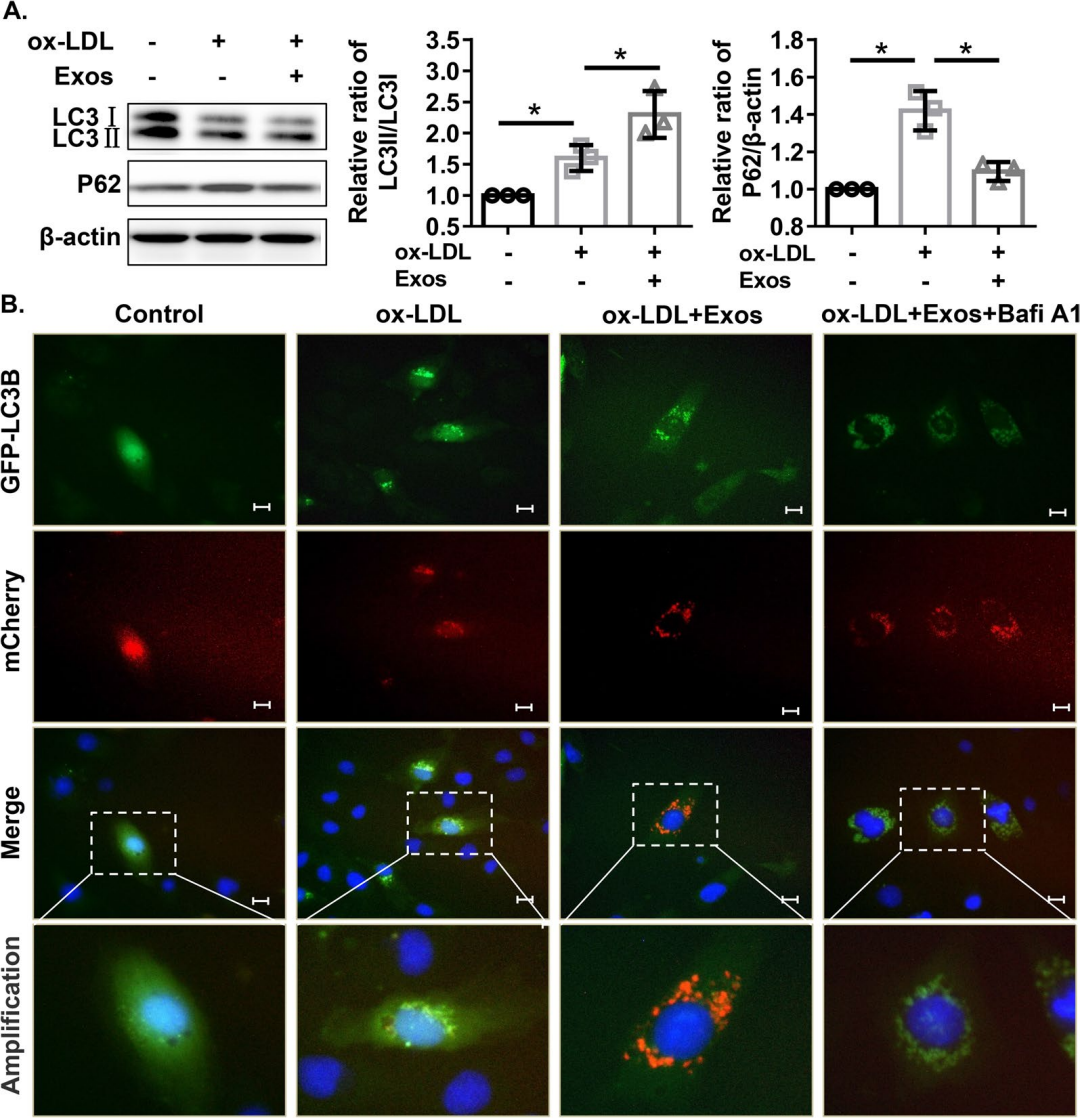
ECFC-derived exosomes rescue autophagic flux in ox-LDL-treated HMECs. **A** Expression of autophagy-related proteins (LC3I, LC3II, and p62) in HMECs after different treatments was detected by western blot assay. Biological replicates = 3, and technical replicates = 1. **B** Representative images of cells infected with Ad-mCherry-GFP-LC3B adenovirus after different treatments. Scale bars = 100 μm. *Exos* ECFCs-exosomes, *Bafi A1* Bafilomycin A1. Biological replicates = 3, and technical replicates = 1. Significant differences between different treatment groups are indicated as *P < 0.05

**Fig. 4:**
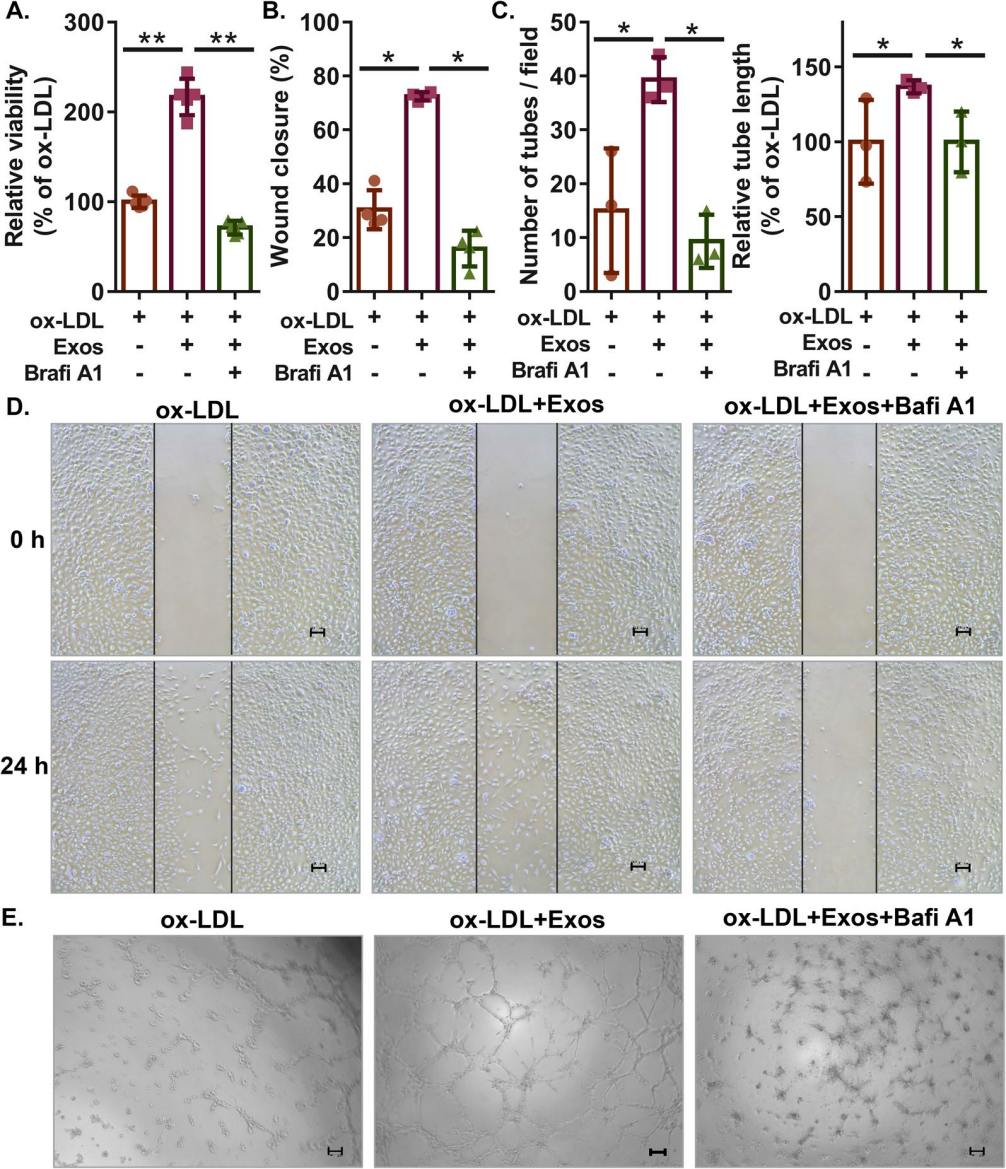
ECFC-derived exosomes improve the proliferation, migration, and tube formation of ox-LDL-treated HMECs in an autophagic flux-dependent manner. **A** The viability of HMECs with different treatments was measured by CCK-8 assay. Biological replicates = 5, and technical replicates = 3. **B, E** Cell migration of HMECs with different treatments was determined by wound healing assay. Scale bars = 100 μm. Biological replicates = 4, and technical replicates = 1. **C, F** Tube formation ability of HMECs with different treatments was evaluated by tube formation assay. Scale bars = 100 μm. *Exos* ECFCs-exosomes. Biological replicates = 3 and technical replicates = 1. Significant differences between different treatment groups are indicated as *P < 0.05 and **P < 0.01

It is well known that autophagy plays an important role in high fat-induced vascular damage or atherosclerosis [30, 31]. To investigate whether ECFC-derived exosomes exert their effects through autophagic pathways, we examined the effects of ECFC-derived exosomes on autophagic flux in HMECs treated with ox-LDL. The results of western blotting showed that the relative ratio of LC3II/LC3I in HMECs was increased in response to ox-LDL treatment, which was further enhanced by ECFC-exosome treatment, and ECFC-exosome treatment significantly attenuated ox-LDL-induced increases in p62 protein levels in HMECs (Fig. 3A). The Ad-mCherry-GFP-LC3B system was next applied to trace different stages of autophagy. The results demonstrated that ox-LDL prevented the formation of autolysosomes with the accumulation of spotty yellow fluorescence (Fig. 3B), and the formation of autolysosomes was rescued by ECFC-exosomes with increased red fluorescence (Fig. 3B), which was abolished by the autophagy inhibitor bafilomycin A1 (Fig. 3B). In vitro functional assays further revealed that bafilomycin A1 treatment significantly abolished the enhanced effects of ECFC-exosomes on cell viability, cell migration and tube formation of HMECs in response to ox-LDL treatment (Fig. 4). These results indicated that ECFC-exosomes suppress ox-LDL-treated HMEC injury in an autophagy-dependent mechanism.

### ECFC-derived miR-21-5p rescues autophagic flux and inhibits endothelial cell injury in ox-LDL-induced HMECs

**Fig. 5:**
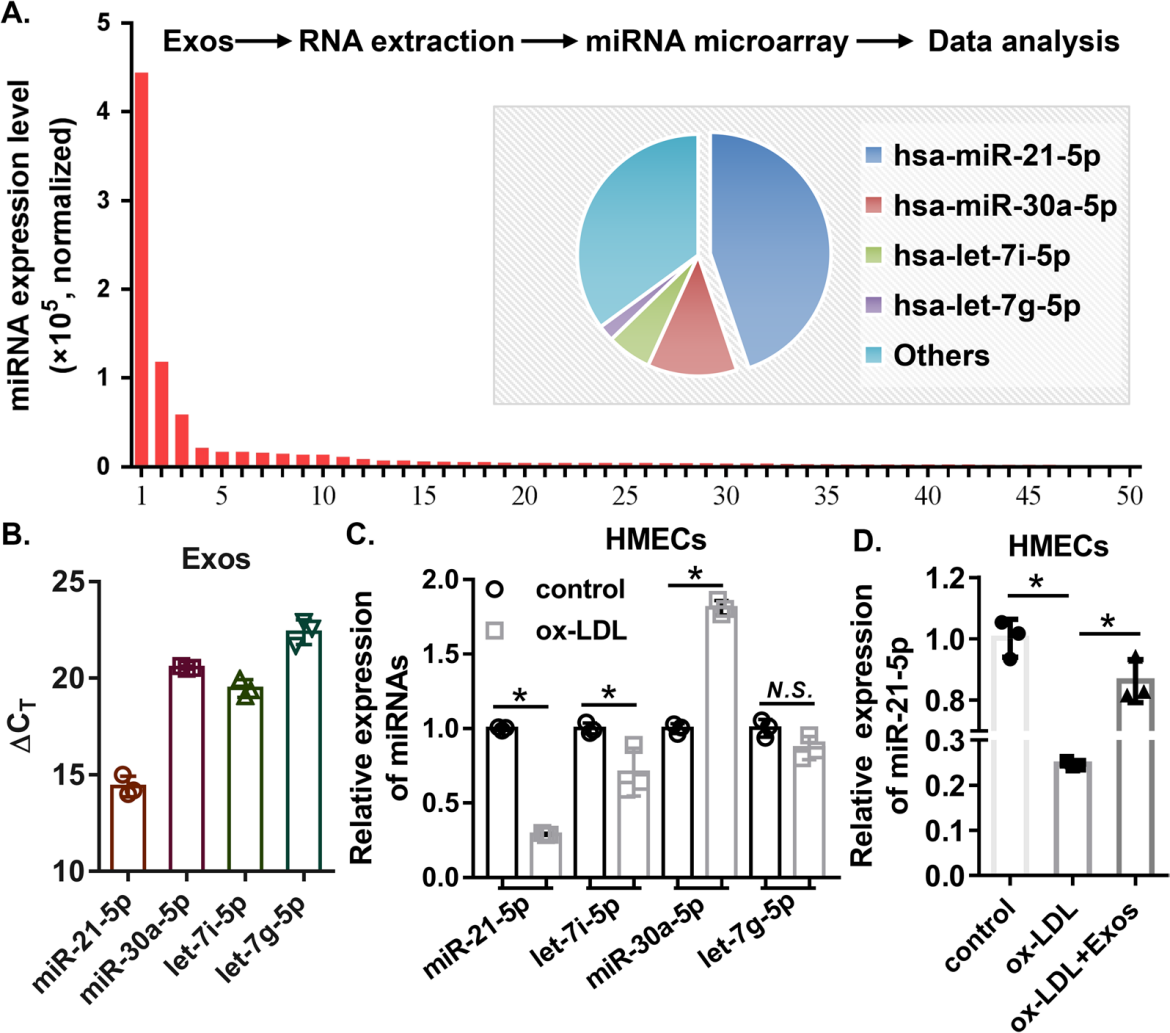
miRNA cargo in ECFCs-exosomes. **A** Top 30 miRNAs expression in ECFCs-exosomes. The pie chart illustrates the miRNA composition of ECFC exosomes. **B** Validation of miRNA expression in ECFC exosomes. Biological replicates = 3, and technical replicates = 3. **C** Altered expression levels of miRNA in HMECs with or without ox-LDL. Biological replicates = 3, and technical replicates = 3. **D** Expression of miR-21-5p was detected by quantitative real-time PCR. *Exos* ECFCs-exosomes. Biological replicates = 3, and technical replicates = 3. *N.S.* not significant; significant differences between different treatment groups are indicated as *P < 0.05

Studies have demonstrated that exosomes can exert their functions in targeted cells via miRNAs [32–34]. Here, we analysed the miRNA expression profiles of ECFC-exosomes using miRNA microarray analysis. The most abundant miRNAs included miR-21-5p, miR-30a-5p, let-7i-5p and let-7g-5p (Fig. 5A), which was further verified by qRT–PCR (Fig. 5B). Furthermore, ox-LDL had no effect on the expression levels of let-7g-5p but significantly suppressed miR-21-5p and let-7i-5p expression levels and increased miR-30a-5p expression levels (Fig. 5C). Among them, miR-21-5p was the most downregulated in HMECs treated with ox-LDL, and miR-21-5p was selected for subsequent experiments. Furthermore, ECFC-exosome treatment largely attenuated the inhibitory effects of ox-LDL treatment on miR-21-5p expression in HMECs (Fig. 5D). These results suggested that ECFC-derived exosomes transmit miR-21-5p to HMECs exposed to ox-LDL.

**Fig. 6:**
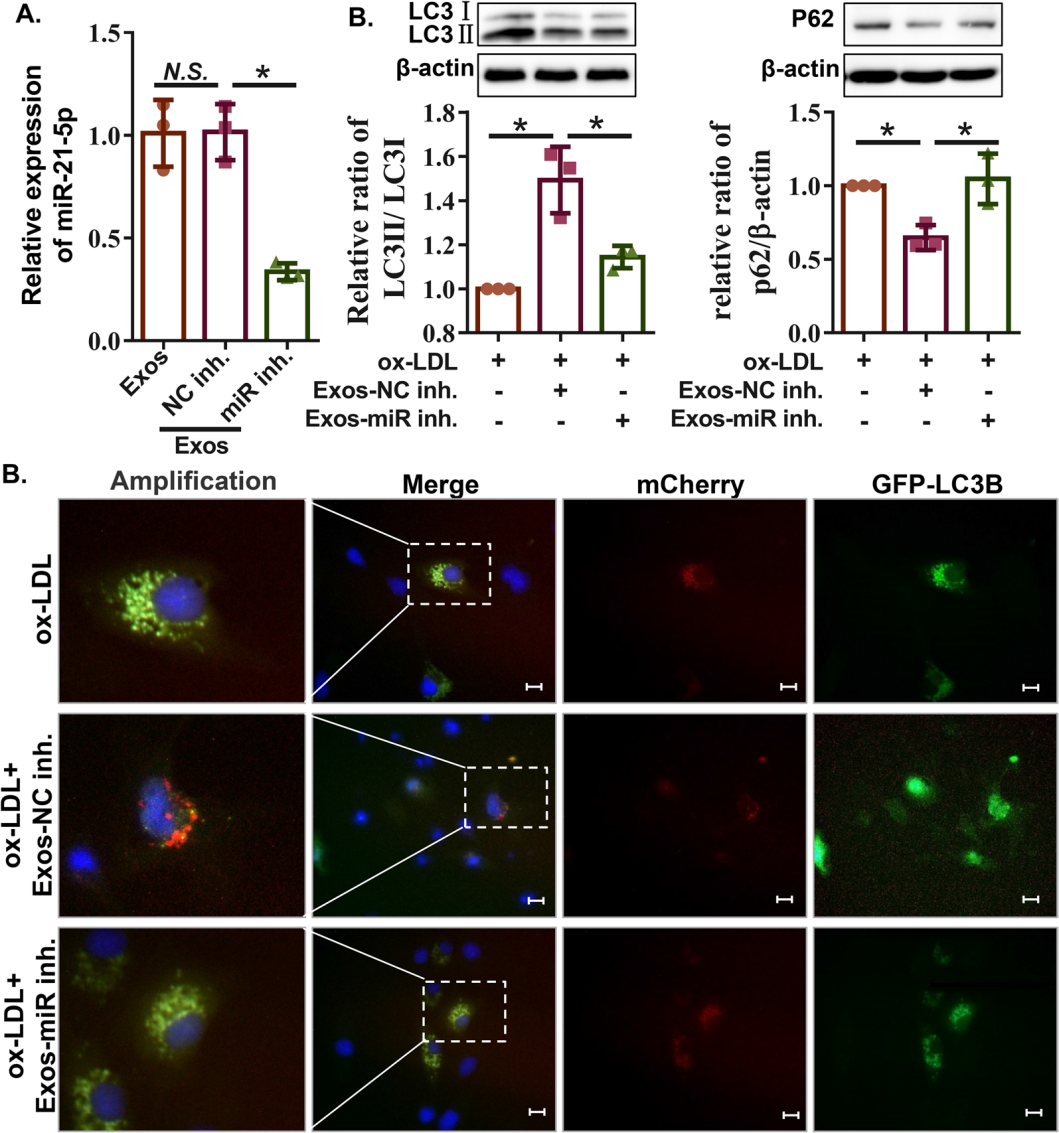
ECFC-derived exosomes rescue autophagic flux in ox-LDL-treated HMECs through miR-21-5p. **A** Expression of miR-21-5p in ECFC exosomes transfected with negative control inhibitor or miR-21-5p inhibitor was detected by qRT–PCR. Biological replicates = 3, and technical replicates = 3. **B** The expression of autophagy-related proteins (LC3I, LC3II, and p62) in HMECs with different treatments was detected by western blot assay. Biological replicates = 3, and technical replicates = 1. **C** Representative images of HMECs transfected with Ad-mCherry-GFP-LC3B adenovirus followed by different treatments. Scale bars = 100 μm. *Exos-miR inh* ECFC-exosomes were transfected with miR-21-5p inhibitor, *Exos-NC inh* ECFC-exosomes were transfected with NC inhibitor. Biological replicates = 3 and technical replicates = 1. Significant differences between different treatment groups are indicated as *P < 0.05

To further determine whether ECFC-exosomes play a protective role in the ox-LDL-induced HMEC injury model by transmitting miR-21-5p, we transfected an miR-21-5p inhibitor or its negative control (NC inhibitor) into ECFC-exosomes using the Exo-fect Exosome Transfection Kit according to the manufacturer’s protocol. The qRT-PCR results suggested that miR-21-5p expression in ECFC-exosomes converted to NC inhibitor was no different from that in ECFC-exosomes, while miR-21-5p expression in ECFC-exosomes converted to miR-21-5p inhibitor was significantly lower than that in ECFC-exosomes converted to NC inhibitor (Fig. 6A), suggesting that the miR-21-5p inhibitor significantly inhibited miR-21-5p expression in ECFC-exosomes. Furthermore, expression levels of miR-21-5p in HMECs with ECFC-exosome-miR inhibitor were significantly lower than that in HMECs without ECFC-exosome-miR inhibitor (Additional file 3: Figure S2A). The results of the CCK-8 assay and LDH release assay revealed that ECFC-exosomes inhibited ox-LDL-induced cell viability decline and LDH release in HMECs, while silencing miR-21-5p ECFC exosomes weakened the effect of ECFC-exosomes on HMECs treated with ox-LDL (Additional file 3: Figure S2B and S2C). Furthermore, it was found in this study that ECFC-exosomes protect against ox-LDL-induced HMEC injury by enhancing autophagy and restoring autophagy flow (Fig. 4B, C). Therefore, we further evaluated whether ECFC-exosomes regulate autophagy or autophagic flux by delivering miR-21-5p. We assessed the protein expression of autophagy markers (LC3II, LC3I and P62) in HMECs with different treatments using western blotting. The LC3II/LC3I ratio and P62 protein expression were significantly increased in ECFC-exosome-treated cells, but the LC3II/LC3I ratio was markedly decreased and the P62 protein expression was significantly increased in the silenced miR-21-5p ECFC-exosome-treated cells (Fig. 6B), indicating that ECFC-exosomes enhance autophagy and repair autophagic flux by delivering miR-21-5p. Consistent with the results of the western blotting analysis, the Ad-mCherry-GFP-LC3B adenovirus infection assay showed that the formation of autolysosomes was increased in ox-LDL-induced HMECs with ECFC-exosome treatment, which was attenuated by miR-21-5p inhibition (Fig. 6C). Taken together, these results revealed that ECFC-derived exosomes protect against ox-LDL-induced HMEC damage by transmitting miR-21-5p to mediate autophagy or autophagic flux.

### ECFC-derived exosomes deliver miR-21-5p to target SIPA1L2 expression in ox-LDL-induced HMECs

**Fig. 7:**
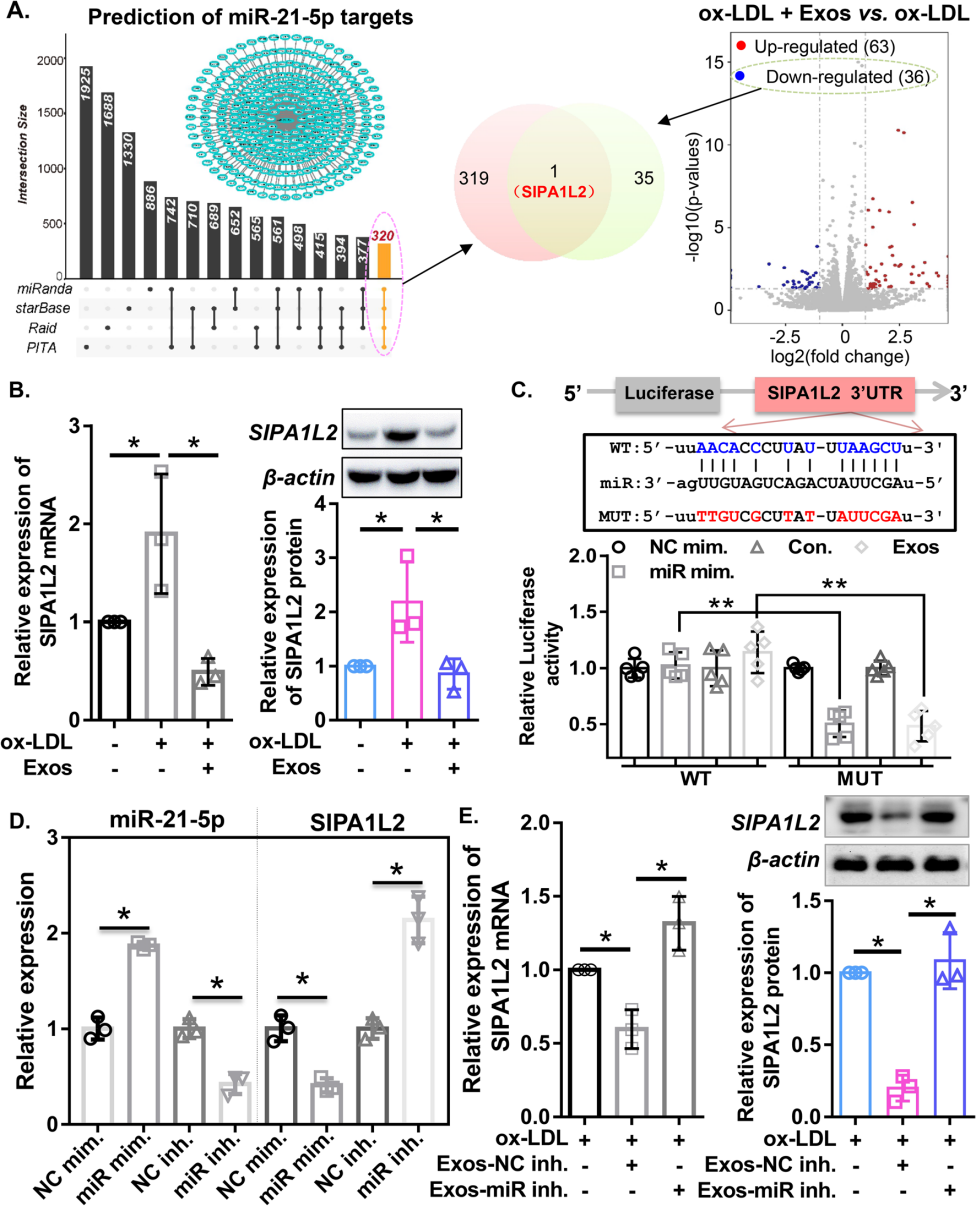
EPC-exosome-derived miR-21-5p targets the 3’UTR of SIPA1L2. **A** Venn diagram illustrating the overlapping miR-21-5p-targeted genes predicted using bioinformatics tools (miRanda, starBase, RAID and PITA) and downregulated genes in HMECs following ECFC-exosome treatment. **B** mRNA and protein expression of SIPA1L2 in HMECs with different treatments was determined by qRT-PCR and western blot assay, respectively. Biological replicates = 3, and technical replicates = 1–3. **C** The interaction between miR-21-5p and the 3’UTR of SIPA1L2 was evaluated using the dual-luciferase reporter assay. Biological replicates = 5, and technical replicates = 1. **D** Expression of miR-21-5p and SIPA1L2 in HMECs transfected with miR-21-5p mimics or inhibitor was determined by qRT-PCR. Biological replicates = 3, and technical replicates = 3. **E** mRNA and protein expression of SIPA1L2 in HMECs with different treatments was determined by qRT-PCR and western blot assay, respectively. *Exos* ECFC-exosomes, *Exos-miR inh* ECFC-exosomes were transfected with miR-21-5p inhibitor, *Exos-NC inh* ECFC-exosomes were transfected with NC inhibitor. Biological replicates = 3, and technical replicates = 1–3. *N.S.* not significant; significant differences between different treatment groups are indicated as *P < 0.05 and **P < 0.01

To determine the downstream targets of miR-21-5p, we first performed bioinformatics analysis using miRanda, starBase, RAID and PITA, and a total of 320 commonly targeted genes were identified (Fig. 7A). Furthermore, we extracted the differentially expressed genes in ECFC-exosomes + ox-LDL- or PBS + ox-LDL-treated HMECs, and a total of 36 downregulated differentially expressed genes were detected. SIPAL1L2 was identified as the common gene between the predicted targets and downregulated DEGs (Fig. 7A). qRT-PCR and western blot assays showed that ox-LDL treatment significantly increased mRNA and protein expression levels of SIPAL1L2, which was significantly attenuated by ECFC-exosome treatment (Fig. 7B).

As predicted by the bioinformatics tools, the binding sites between miR-21-5p and the SIPAL1L2 3’UTR are shown in Fig. 7C. The dual-luciferase reporter assay showed that miR-21-5p mimics and ECFC-exosome treatment both significantly inhibited the luciferase reporter activity of SIPAL1L2 3’UTR-wt but not SIPAL1L2 3’UTR-mut (Fig. 7C). To investigate the regulatory effect of miR-21-5p on SIPA1L2, miR-21-5p mimics or inhibitors were transfected into HMECs. The qRT–PCR results indicated that miR-21-5p expression was significantly increased and SIPA1L2 expression was markedly decreased in HMECs transfected with miR-21-5p mimics (Fig. 7D), indicating that miR-21-5p negatively regulates SIPA1L2 expression. Furthermore, the qRT–PCR and western blotting results confirmed that ECFC-exosomes suppressed mRNA and protein expression levels of SIPA1L2 in ox-LDL-induced HMECs, which was significantly reversed by miR-21-5p-mediated inhibition (Fig. 7E). Taken together, these results illustrate that SIPA1L2 is the target gene of miR-21-5p, and ECFC-exosomes regulate SIPA1L2 expression by delivering miR-21-5p to ox-LDL-induced HMECs.

To explore the effect of SIPA1L2 on autophagy and autophagic flux in ox-LDL-induced HMECs, HMECs were transfected with three shRNAs specifically targeting SIPA1L2 (SIPA1L2 shRNA#1, #2, and #3) to restrain SIPA1L3 expression. SIPA1L2 shRNA#1 (sh-SIPA1L2) was observed to have the optimum inhibitory efficiency (Additional file 4: Figure S3A) and was used in subsequent studies. The qRT-PCR results showed that treatment with ox-LDL for 24 h significantly increased SIPA1L2 mRNA expression, and this effect was abolished by shRNA-mediated silencing of SIPA1L2 in HMECs (Additional file 4: Figure S3B), suggesting that transfection of sh-SIPA1L2 with HMECs effectively inhibits SIPA1L2 expression induced by ox-LDL. Furthermore, western blotting was performed to estimate the protein expression levels of autophagy-related genes (Beclin 1, LC3I, LC3II, and P62), and the results showed that ox-LDL treatment significantly increased the protein expression of Beclin-1 and P62 and promoted the turnover of LC3II in HMECs (Additional file 4: Figure S3C-D), indicating that ox-LDL induces autophagic flux dysfunction in HMECs. The downregulation of SIPA1L2 markedly enhanced Beclin-1 protein expression and promoted the turnover of LC3II and P62 degradation in ox-LDL-treated HMECs (Additional file 4: Figure S3C-D), suggesting that downregulation of SIPA1L2 improves ox-LDL-induced HMEC autophagic flux dysfunction and autophagic activity. Furthermore, the autophagy inhibitor Bafi A1 was used to verify autophagic flux dysfunction and the autophagic activation effect of SIPA1L2 downregulation in ox-LDL-treated HMECs. The results revealed that costimulation of HMECs with sh-SIPA1L2 and Bafi A1 significantly decreased the protein expression levels of autophagy-related genes (Beclin1 and p62) and inhibited the turnover of LC3II compared to those after treatment with sh-SIPA1L2 alone (Additional file 4: Figure S3E-F). Moreover, the CCK-8 assay results showed that SIPA1L2 suppression with sh-SIPA1L2 enhanced cell proliferation and that Bafi A1 treatment alone reduced cell proliferation in ox-LDL-treated HMECs, which was reversed when the cells were costimulated with sh-SIPA1L2 and Bafi A1 (Additional file 4:Figure S3G). These data suggested that silencing SIPA1L2 in ox-LDL-treated HMECs promotes cell proliferation by enhancing autophagy and repairing autophagic flux dysfunction.

### ECFC-exosomes promote endothelial repair and activate autophagy by delivering miR-21-5p in an atherogenic rat model of vascular injury

**Fig. 8:**
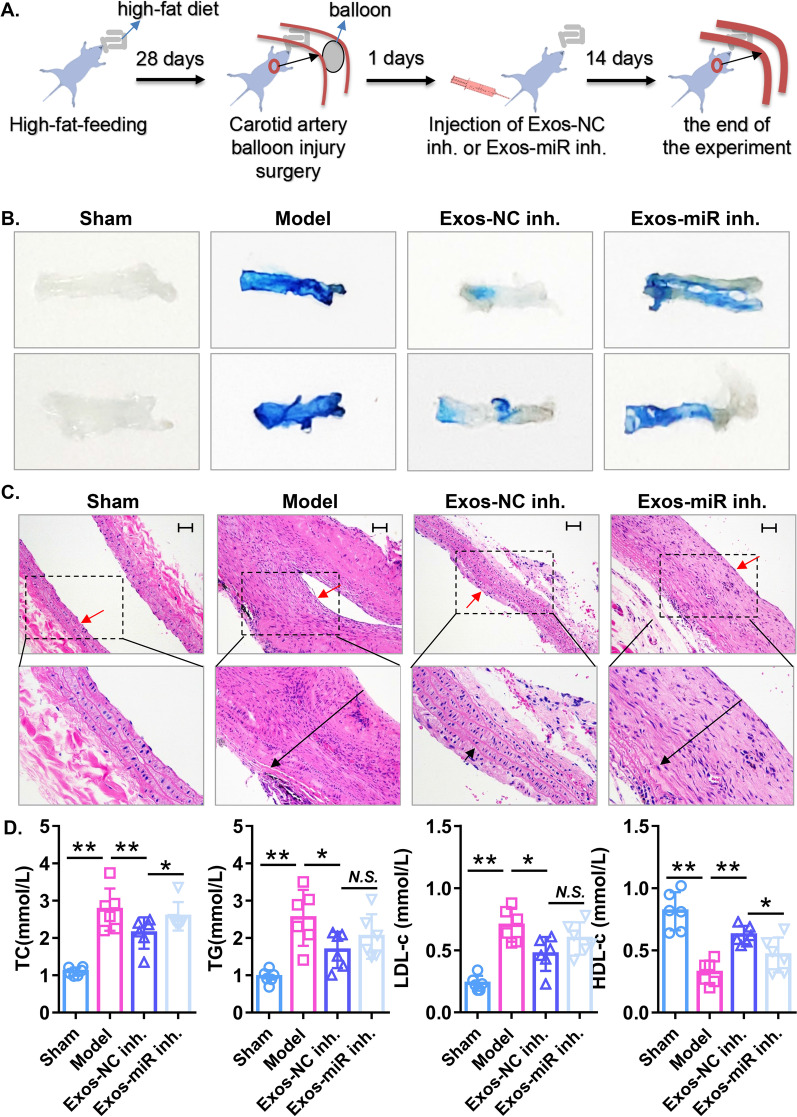
ECFC-derived exosomes promote reendothelialization and rescue intimal hyperplasia in a rat atherosclerosis model in a miR-21-5p-dependent manner. **A** A flowchart illustrating experiments to evaluate the therapeutic effects of ECFC-exosomes in a rat atherosclerosis model. **B** The injured areas of carotid arteries in rats with different treatments were evaluated using Evans blue staining. Biological replicates = 6, and technical replicates = 1. **C** Morphology of the carotid arteries in the rats with different treatments was evaluated by HE staining. The red arrows indicate the endothelial layers, and the black arrows indicate intimal hyperplasia. Biological replicates = 6, and technical replicates = 1. **D** Concentrations of TC, TG, LDL-c, and HDL-c in serum samples at the end of the in vivo experiment. Sham, control rat; Model, high-fat diet combined with balloon injury to construct atherosclerotic rat model of vascular injury; *Exos-miR inh* atherosclerotic rat model of vascular injury treated with ECFCs-exosomes transfected with miR-21-5p inhibitor, *Exos-NC inh* atherosclerotic rat model of vascular injury treated with ECFCs-exosomes transfected with NC inhibitor. Biological replicates = 6, and technical replicates = 3. Significant differences between different treatment groups are indicated as *P < 0.05 and **P < 0.01

**Fig. 9:**
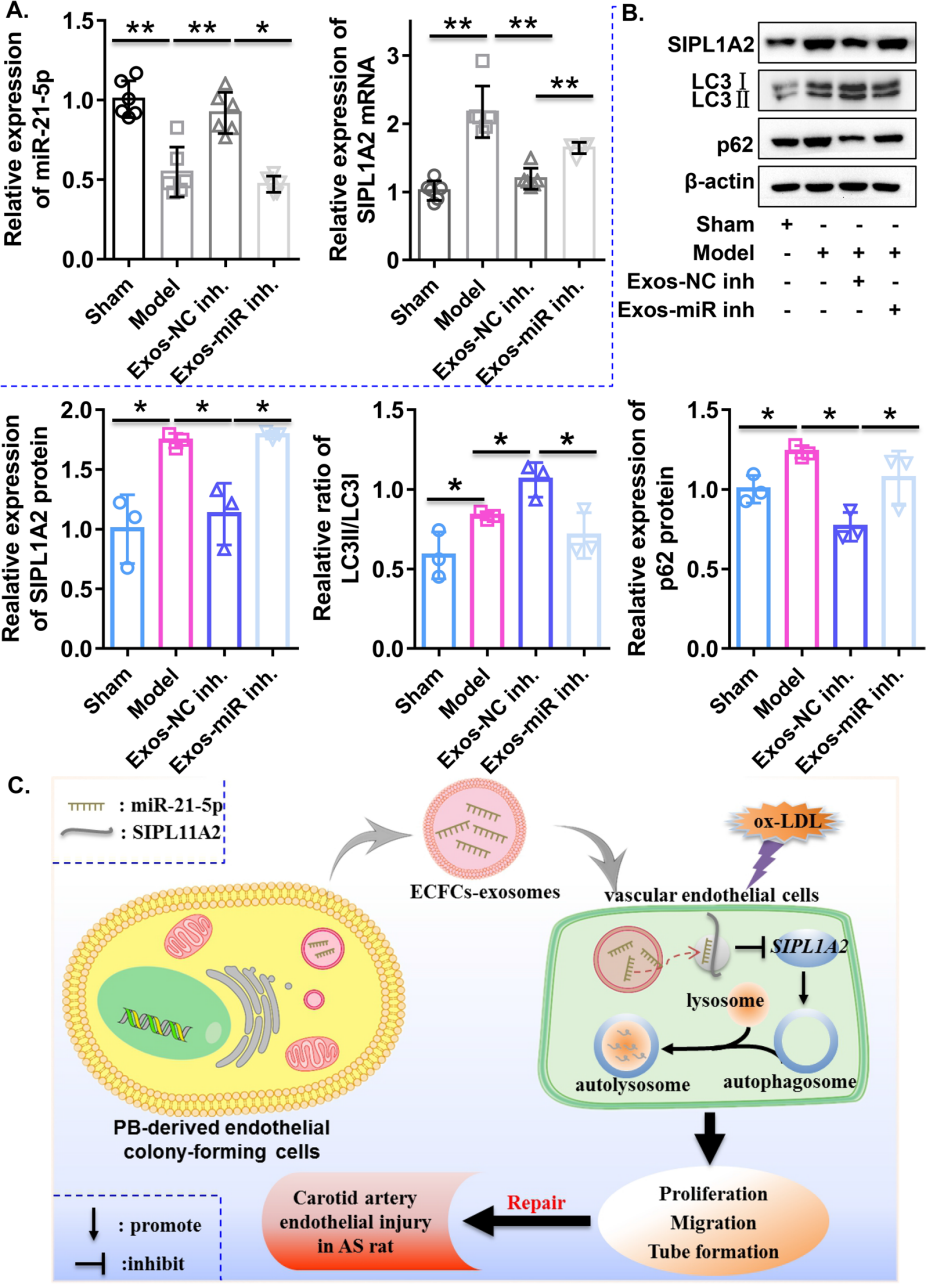
ECFC-derived exosomes repair vascular injury by rescuing autophagic flux through the miR-21-5p/SIPA1L2 axis in a rat atherosclerosis model. **A** Expression levels of miR-21-5p and SIPA1L2 in rats with different treatments were determined by qRT–PCR. Biological replicates = 3–6, and technical replicates = 3. **B** Protein levels of SIPA1L2 and autophagy-related proteins (LC3I, LC3II and p62) in rats with different treatments were determined by qRT-PCR and western blot assay. Biological replicates = 3, and technical replicates = 1. **C** The schematic diagram illustrates that ECFC-exosomes repair vascular injury by rescuing autophagic flux through the miR-21-5p/SIPA1L2 axis in a rat atherosclerosis model. *Exos-miR inh* ECFC-exosomes transfected with miR-21-5p inhibitor, *Exos-NC inh* ECFC-exosomes transfected with NC inhibitor, *N.S.* not significant; significant differences between different treatment groups are indicated as *P < 0.05 and **P < 0.01

To further evaluate the role and underlying mechanism of ECFC-exosomes in endothelial repair in vivo, we constructed an atherogenic rat model of vascular injury using a high-fat diet combined with balloon injury. The in vivo experimental procedures are illustrated in Fig. 8A. After 4 weeks of high-fat diet treatment, serum levels of TC, TG, and LDL-c were elevated, and serum levels of HDL-c were decreased in rats (Additional file 5: Figure S4), indicating that the hyperlipidaemia or atherosclerosis rat model had been successfully established. After carotid artery balloon injury in hyperlipidaemic rats for 14 days, Evans blue staining indicated that rats in the model groups exhibited serious vascular endothelial injury, and ECFC-exosome treatment alleviated vascular endothelial injury in the atherogenic rat model of vascular injury, which was significantly reversed by treatment with ECFC-exosomes with miR-21-5p knockdown (Fig. 8B). Intimal hyperplasia is the primary symptom of atherosclerosis or PTAC after surgery. We evaluated levels of intimal hyperplasia by haematoxylin and eosin (HE) staining, and our results demonstrated severe intimal hyperplasia in the atherogenic rat model of vascular injury, while administration of ECFC-exosomes largely prevented intimal hyperplasia, which was hindered by treatment with ECFC-exosomes with miR-21-5p knockdown (Fig. 8C). Interestingly, we found that ECFC-exosome treatment significantly decreased serum levels of TC, TG and LDL-c but increased serum levels of HDL-c in the atherogenic rat model of vascular injury, and ECFC-exosomes with miR-21-5p knockdown treatment partially weakened the beneficial regulation of ECFC-exosomes on lipid indices (Fig. 8D). These data suggested that ECFC-exosomes might regulate lipid homeostasis to inhibit vascular injury in an atherogenic rat model of vascular injury, and the regulatory mechanism is partly dependent on miR-21-5p transmitted by ECFC-exosomes. In addition, compared to the sham group, miR-21-5p expression was significantly downregulated and mRNA and protein expression of SIPL1A2 was significantly upregulated in the model group. ECFC-exosome treatment significantly increased miR-21-5p and decreased SIPL1A2 mRNA expression in the rat model, which was significantly attenuated by ECFC-exosomes with miR-21-5p knockdown (Fig. 9A), suggesting that ECFC-exosomes regulate the mRNA and protein expression of SIPL1A2 in the atherogenic rat model of vascular injury through miR-21-5p transmission. Furthermore, western blotting results showed that protein levels of the LC3II/LC3I ratio and p62 were upregulated in the model group, while ECFC-exosomes treatment significantly decreased the p62 protein expression but increased the ratio of LC3II/LC3I in the rat model, which was largely reversed by ECFC-exosomes with miR-21-5p knockdown (Fig. 9B). Taken together, these results indicated that ECFC exosomes repair vascular endothelial injury and enhance autophagy by delivering exosomal miR-21-5p in an atherogenic rat model of vascular injury.

## Discussion

ECFCs have been shown to promote the formation of new endothelium in animal models, in which vessel injury occurs after balloon injury, myocardial infarction, or coronary microembolization [35–37]. Exosomes have emerged as an important paracrine mechanism of cell-to-cell communication by facilitating the transfer of RNAs or proteins from one cell to a recipient cell [38], and their use is currently considered to represent a promising alternative to stem cell therapy [39]. Studies have revealed that ECFC-exosomes promote endothelial cell repair in rat models of balloon injury [23, 24]. However, it is still unknown whether ECFC exosomes protect against endothelial injury in AS. In this study, we first found that ECFC-exosomes protected against ox-LDL-induced vascular endothelial injury by repairing autophagic flux. Subsequently, we demonstrated that miR-21-5p, which is abundant in ECFC exosomes, binds to the 3’-UTR of SIPA1L2 to inhibit its expression and enhance autophagic flux, and ECFC-exosome knockout of miR-21-5p reversed ECFC-exosome-decreased SIPA1L2 expression in ox-LDL-induced vascular injury. Finally, our results revealed that ECFC-exosomes repaired vascular endothelial injury, regulated lipid balance and activated autophagy in the atherogenic rat model of vascular injury, whereas these effects were eliminated with ECFC-exosomes with knockdown of miR-21-5p.

Autologous stem cells from patients are difficult to acquire and often yield low quality cells [40]. Allogeneic stem cells, on the other hand, have limited applications due to their immunogenicity [41]. To overcome these problems, researchers have focused on the paracrine products of stem cells. Several studies have demonstrated that ECFC-exosomes could be used to treat certain vascular diseases in different tissues [13–15]. Recent studies have shown that autophagy and autophagic flux play key roles in cardiovascular diseases, including atherosclerosis [42]. Impaired autophagic flux or inhibition of autophagic flux aggravates ox-LDL-induced inflammatory responses in endothelial cells, whereas enhancing autophagic flux alleviates ox-LDL-induced inflammatory responses [43]. miR-100 suppresses vascular injury by stimulating endothelial autophagy [44]. Shimaa et al. [45] reported that EPCs confer therapeutic effects against epilepsy by upregulating autophagy. However, the effect of ECFC-exosomes on autophagy or autophagic flux in ox-LDL-induced vascular endothelial injury remains unclear. In the present study, our results demonstrated that ECFC-exosomes alleviate ox-LDL-induced vascular endothelial injury and reverse the impaired autophagic flux induced by ox-LDL, whereas these effects were eliminated with the autophagy inhibitor bafilomycin A1, suggesting that ECFC-exosomes protect against ox-LDL-induced vascular endothelial injury by enhancing autophagic flux. Furthermore, we found that ECFC-exosomes promote vascular reendothelialization and inhibit intimal hyperplasia in an atherogenic rat model of vascular injury, consistent with the results of previous studies. Li et al. [23] and Hu et al. [16] reported that exosomes of EPCs derived from human umbilical cord blood promote reendothelialization and inhibit neointimal formation in rat models of balloon injury by upregulating endothelial cell function. The latest research showed that exosomes of EPCs derived from mouse bone marrow ameliorated endothelial dysfunction and decreased lipid droplets in thoracic aortas in a mouse model of diabetes [46]. Interestingly, our results also demonstrated that ECFC exosomes activate autophagy and regulate lipid metabolism homeostasis in an atherogenic rat model of vascular injury, which may be the potential mechanism by which ECFC exosomes promote the repair of injured vasculature.

Previous studies have demonstrated that ECFC-exosome-mediated transfer of miRNAs closely participates in ECFC-exosome-mediated e recovery from several diseases. For example, EPC-exosomes ameliorate lipopolysaccharide-induced acute lung injury and improve sepsis outcomes by delivering miR-126 [47–49]. Moreover, EPC-exosomes promote proliferation, migration and tube formation via the delivery of miR-21-5p in endothelial cells [24]. Therefore, we speculate that ECFC-exosomes protect against vascular endothelial injury under hyperlipidaemic conditions by delivering one or more miRNAs. We extracted total RNA from ECFC-exosomes and analysed the components of the miRNAs using miRNA microarray analysis. Our results indicated that miR-21-5p was the most highly expressed in ECFC-exosomes, consistent with the sequencing results of ECFC-exosomes from other origins [24, 46], indicating that EPCs from different origins may share a similar constitution. We further verified by qRT-PCR that miR-21-5p exhibited the highest abundance in ECFC-exosomes, consistent with the miRNA microarray. Moreover, our data revealed for the first time that ECFC exosomes inhibit vascular endothelial cell damage by delivering exosomal miR-21-5p to recipient cells in an AS model induced by ox-LDL in vitro and an atherogenic rat model of vascular injury in vivo. Similarly, many studies have revealed the protective function of exosomal miR-21-5p in different diseases [50–53], suggesting that exosome-mediated transfer of miR-21-5p is an important mechanism for cell-to-cell communication and the regulation of recipient cell functions. Interestingly, our results indicated that ECFC-exosomes enhance autophagic flux and regulate lipid metabolism balance by delivering exosomal miR-21-5p. Previous studies have shown that miR-21-5p is involved in the regulation of autophagy, such as the knockout of miR-21-5p to inhibit arsenate-induced autophagy [54] and overexpression of miR-21-5p to induce autophagy in female germline stem cells [55]. In addition, overexpression of miR-21-5p correlated with a less atherogenic lipid profile and decreased serum lipid levels [56, 57].

Studies have shown that miRNAs directly interact with the 3’UTR of their target mRNAs and regulate posttranscriptional genes by blocking translation or degradation of target mRNAs to influence the biological function of cells [58]. In this study, our results demonstrated for the first time that SIPA1L2 was the target of ECFC-exosomal miRNA-21-5p and that ECFC-exosome-mediated transfer of miR-21-5p suppressed SIPA1L2 expression in in vitro and in vivo models. SIPA1L2, also known as SPAR2, is a regulator of Rap1, a member of the SIPA1L family with RapGAP activity [59]. At present, SIPA1L2 has primarily been investigated in the neurological field, and it has been demonstrated that SIPA1L2 interacts with the autophagy marker LC3 [60], indicating that SIPA1L2 is associated with the autophagic pathway. Our data also demonstrated that silencing SIPA1L2 in ox-LDL-treated HMECs promoted cell proliferation by enhancing autophagy and repairing autophagic flux dysfunction.

Despite these promising findings, this study has several limitations. First, the present study lacked evidence that the exosomes administered in vivo reached the injury site, which may still require further investigation. Second, the role of *SIPA2L1* in atherosclerosis progression has not been further verified in vivo. Third, the underlying mechanism of SIPA1L2 in ox-LDL-induced autophagic flux dysfunction remains largely unknown. Fourth, a rescue experiment was not performed to verify that ECFC-exosomes or miR-21-5p repair vascular injury in a high-fat rat balloon injury model by regulating SIPA1L2-mediated autophagy. Fifth, this study focused on the effect of ECFC exosomes on atherosclerosis-induced endothelial cell injury, but ECFC-exosomes on smooth muscle cells were not explored. These points will be addressed in future studies.

## Conclusion

In summary, our study demonstrated that ECFC-exosomes protect against atherosclerosis- or PTCA-induced vascular injury by rescuing autophagic flux and inhibiting SIAP1L2 expression by delivering miR-21-5p (Fig. 9C). Our study provides new insight into the molecular mechanism of atherosclerosis-induced vascular intimal injury and facilitates a new therapeutic strategy for repairing vascular endothelial injuries.

## Supplementary Information

**Additional file 1: Table S1.** Sequence of primers for real-time PCR.

**Additional file 2. Figure S1**. Schematic diagram of biological sample processing before miRNA and mRNA expression profiling.

**Additional file 3. Figure S2**. ECFC-exosomes rescue autophagic flux in ox-LDL-treated HMECs through miR-21-5p. **(A)** The expression of miR-21-5p in HMECs with different treatments was detected by qRT–PCR. Biological replicates = 3, and technical replicates = 3. **(B)** The viability of HMECs with different treatments was detected by CCK-8 assay. Biological replicates = 5, and technical replicates = 3. **(C)** LDH release of HMECs with different treatments was examined using LDH release assay. Biological replicates = 5, and technical replicates = 3. *N.S.* = not significant; significant differences between different treatment groups are indicated as *P < 0.05 and **P < 0.01.

**Additional file 4. Figure S3**. Silencing SIPA1L2 mediates autophagy to protect against ox-LDL-induced HMEC injury. **(A)**. Expression of SIPA1L2 in HMECs transfected with negative control shRNA or SIPA1L2 shRNAs was detected by qRT–PCR. Biological replicates = 3, and technical replicates = 3. **(B)** RT–PCR showing that SIPA1L2 shRNA#1 (sh-SIPA1L2) inhibits the mRNA expression of SIPA1L2 in ox-LDL-treated HMECs. Biological replicates = 3, and technical replicates = 3. **(C-D)** Protein expression of autophagy-related genes in HMECs treated with ox-LDL or sh-SIPA1L2 was detected by western blot. Biological replicates = 3, and technical replicates = 1. **(E–F)** Protein expression of autophagy-related genes in HMECs treated with ox-LDL, sh-SIPA1L2 or Bafi A1 was detected by western blot. Biological replicates = 3, and technical replicates = 1. **(G)** The viability of HMECs with different treatments was detected by CCK-8 assay. Biological replicates = 5, and technical replicates = 3. *N.S.* = not significant; significant differences between different treatment groups are indicated as *P < 0.05 and **P < 0.01.

**Additional file 5. Figure S4**. Concentrations of TC, TG, LDL-c, and HDL-c in serum from rats after a 4-week high-fat diet. Sham, control rat; Model, high-fat diet combined with balloon injury to construct atherosclerotic rat model of vascular injury; Exos-miR inh, atherosclerotic rat model of vascular injury treated with ECFCs-exosomes transfected with miR-21-5p inhibitor; Exos-NC inh, atherosclerotic rat model of vascular injury treated with ECFCs-exosomes transfected with NC inhibitor. Biological replicates = 6, and technical replicates = 3. *N.S.* = not significant; significant differences between different treatment groups are indicated as **P < 0.01

## Data Availability

The datasets used and/or analysed in the current study are available from the corresponding author on reasonable request.

## Abbreviations

3’UTR: 3’ Untranslated region
ac-LDL: Acetylated low-density lipoprotein
BSA: Bovine serum albumin
DAPI: 4,6-Diamidino-2-phenylindole
ELISA: Enzyme-linked immunosorbent assay
EPCs: Endothelial progenitor cells
ECFC-Exos: Endothelial colony-forming cell-derived exosomes
FBS: Foetal bovine serum
FCs: Fold changes
FDRs: False discovery rates
HMECs: Human microvascular endothelial cells
LDH: Lactate dehydrogenase
LDL-c: Low-density lipoprotein cholesterol
MUT: Mutant
PBS: Phosphate-buffered saline
PTCA: Percutaneous transluminal coronary angioplasty
qRT–PCR: Quantitative real-time PCR
RMA: Robust multiarray average
SD: Standard deviation
TEM: Transmission electron microscopy
TG: Triglyceride
UCB: Umbilical cord blood
UEA-l: Ulex europaeus agglutinin-1
WT: Wild-type
