# Supplementary material for: Endothelial colony-forming cell-derived exosomal miR-21-5p regulates autophagic flux to promote vascular endothelial repair by inhibiting SIPL1A2 in atherosclerosis
Source: Cell Commun Signal. 2022 Mar 12;20:30. doi: 10.1186/s12964-022-00828-0 (PMC8917727; doi:10.1186/s12964-022-00828-0)
Supplement: Supplementary file 2 — Additional file 1: Table S1. Sequence of primers for real-time PCR. [file 12964_2022_828_MOESM2_ESM.docx]

**Table S1.** The sequences were used in the study

| **Name** | **Sequences** |
| --- | --- |
| SIPA1L2 Forward primer | TCGGAAGGAAAAACAAGACAAG |
| SIPA1L2 Reverse primer | TTTGTCGATGGTGGTGAAAAAC |
| GADPH Forward primer | AAGTATGACAACAGCCTCAAG |
| GADPH Reverse primer | TCCACGATACCAAAGTTGTC |
| miR-21-5p Forward primer | GCGCGTAGCTTATCAGACTGA |
| miR-21-5p Reverse primer | AGTGCAGGGTCCGAGGTATT |
| U6-snRNA Forward primer | CTCGCTTCGGCAGCACATATACT |
| U6-snRNA Reverse primer | ACGCTTCACGAATTTGCGTGTC |
| miR-21-5p mimics | UAGCUUAUCAGACUGAUGUUGA |
| NC mimics | UUGUACUACACAAAAGUACUG |
| miR-21-5p inhibitor | UCAACAUCAGUCUGAUAAGCUA |
| NC inhibitor | CAGUACUUUUGUGUAGUACAA |
| SIPA1L2 shRNA#1 | CACCGCAATAGTGATGTCACTATCACGAATGATAGTGACATCACTATTGC |
| SIPA1L2 shRNA#2 | CACCGCATCTCAGGATTAGATTATGCGAACATAATCTAATCCTGAGATGC |
| SIPA1L2 shRNA#3 | CACCGGGCCTATTATTACCGCAAATCGAAATTTGCGGTAATAATAGGCCC |
| NC shRNA | CACCCCTAAGGTTAAGTCGCCCTCGCGAACGAGGGCGACTTAACCTTAGG |
