# Supplementary figures and images for: Endothelial colony-forming cell-derived exosomal miR-21-5p regulates autophagic flux to promote vascular endothelial repair by inhibiting SIPL1A2 in atherosclerosis
Source: Cell Commun Signal. 2022 Mar 12;20:30. doi: 10.1186/s12964-022-00828-0 (PMC8917727; doi:10.1186/s12964-022-00828-0)

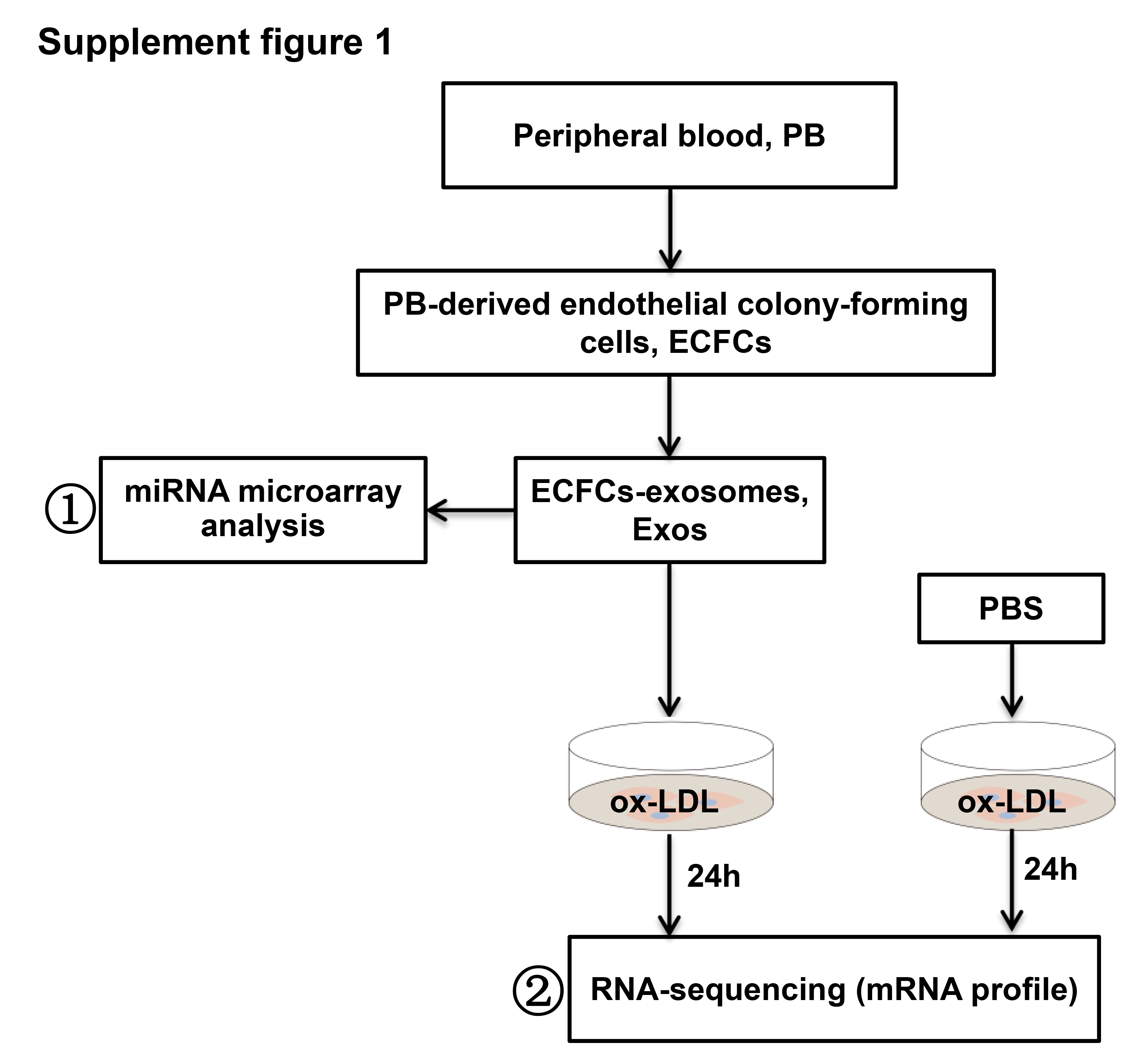

Supplement: Supplementary file 3 — Additional file 2. Figure S1. Schematic diagram of biological sample processing before miRNA and mRNA expression profiling. [file 12964_2022_828_MOESM3_ESM.tif]

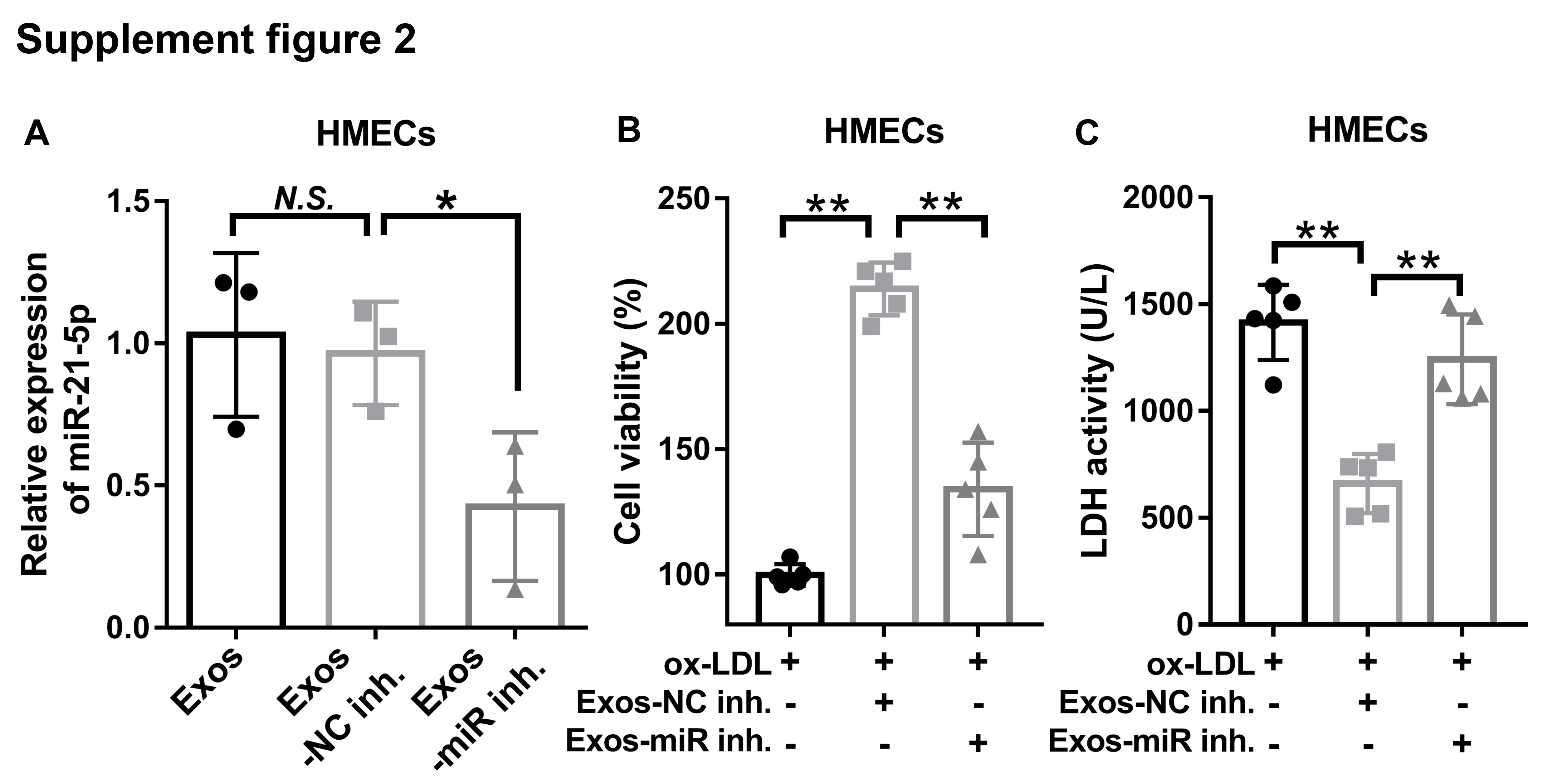

Supplement: Supplementary file 4 — Additional file 3. Figure S2. ECFC-exosomes rescue autophagic flux in ox-LDL-treated HMECs through miR-21-5p. (A) The expression of miR-21-5p in HMECs with different treatments was detected by qRT–PCR. Biological replicates = 3, and technical replicates = 3. (B) The viability of HMECs with different treatments was detected by CCK-8 assay. Biological replicates = 5, and technical replicates = 3. (C) LDH release of HMECs with different treatments was examined using LDH release assay. Biological replicates = 5, and technical replicates = 3. N.S. = not significant; significant differences between different treatment groups are indicated as *P < 0.05 and **P < 0.01. [file 12964_2022_828_MOESM4_ESM.tif]

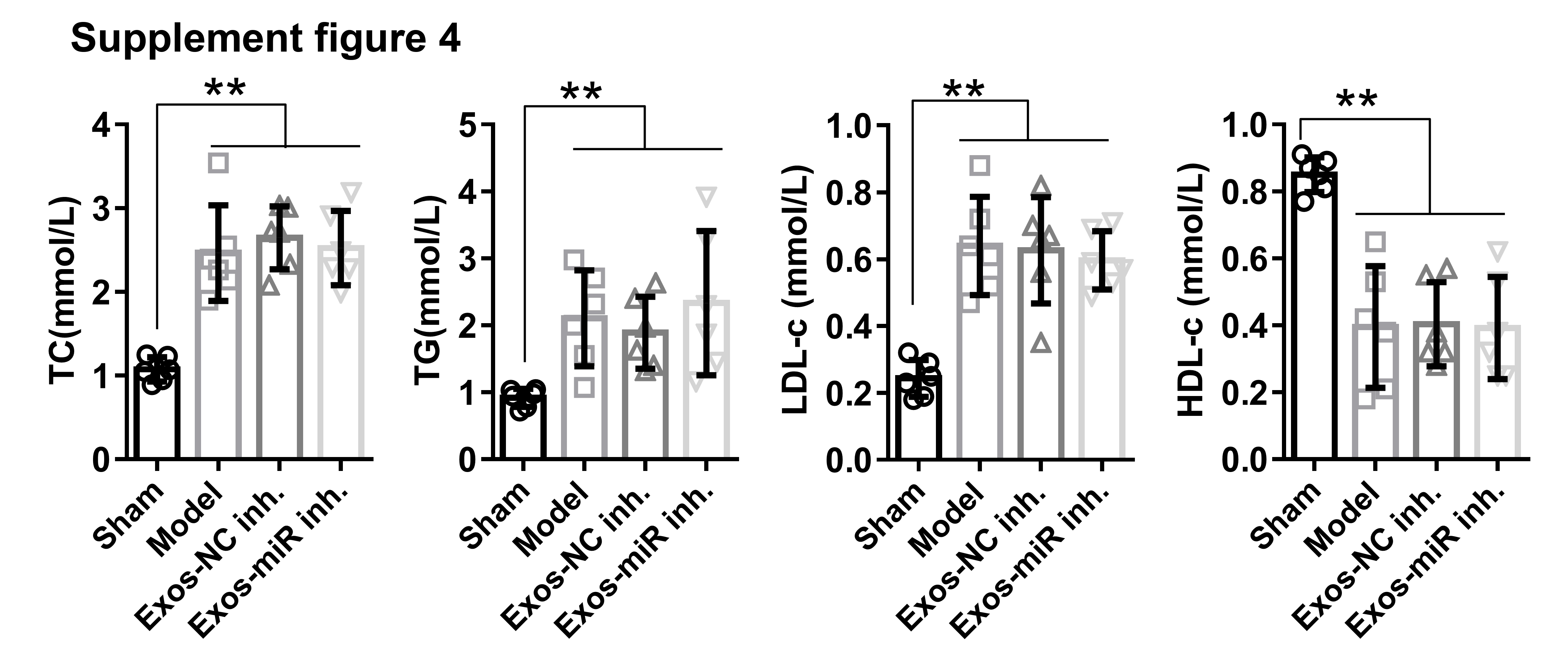

Supplement: Supplementary file 6 — Additional file 5. Figure S4. Concentrations of TC, TG, LDL-c, and HDL-c in serum from rats after a 4-week high-fat diet. Sham, control rat; Model, high-fat diet combined with balloon injury to construct atherosclerotic rat model of vascular injury; Exos-miR inh, atherosclerotic rat model of vascular injury treated with ECFCs-exosomes transfected with miR-21-5p inhibitor; Exos-NC inh, atherosclerotic rat model of vascular injury treated with ECFCs-exosomes transfected with NC inhibitor. Biological replicates = 6, and technical replicates = 3. N.S. = not significant; significant differences between different treatment groups are indicated as **P < 0.01 [file 12964_2022_828_MOESM6_ESM.tif]
